# Methylation of transcription factor YY2 regulates its transcriptional activity and cell proliferation

**DOI:** 10.1038/celldisc.2017.35

**Published:** 2017-10-03

**Authors:** Xiao-nan Wu, Tao-tao Shi, Yao-hui He, Fei-fei Wang, Rui Sang, Jian-cheng Ding, Wen-juan Zhang, Xing-yi Shu, Hai-feng Shen, Jia Yi, Xiang Gao, Wen Liu

**Affiliations:** 1 School of Pharmaceutical Sciences, Fujian Provincial Key Laboratory of Innovative Drug Target Research, Xiamen University, Fujian, China

**Keywords:** YY2, SET7/9, LSD1, lysine methylation, cell proliferation

## Abstract

Yin Yang 1 (YY1) is a multifunctional DNA-binding transcription factor shown to be critical in a variety of biological processes, and its activity and function have been shown to be regulated by multitude of mechanisms, which include but are not limited to post-translational modifications (PTMs), its associated proteins and cellular localization. YY2, the paralog of YY1 in mouse and human, has been proposed to function redundantly or oppositely in a context-specific manner compared with YY1. Despite its functional importance, how YY2’s DNA-binding activity and function are regulated, particularly by PTMs, remains completely unknown. Here we report the first PTM with functional characterization on YY2, namely lysine 247 monomethylation (K247me1), which was found to be dynamically regulated by SET7/9 and LSD1 both *in vitro* and in cultured cells. Functional study revealed that SET7/9-mediated YY2 methylation regulated its DNA-binding activity *in vitro* and in association with chromatin examined by chromatin immunoprecipitation coupled with sequencing (ChIP-seq) in cultured cells. Knockout of YY2, SET7/9 or LSD1 by CRISPR (clustered, regularly interspaced, short palindromic repeats)/Cas9-mediated gene editing followed by RNA sequencing (RNA-seq) revealed that a subset of genes was positively regulated by YY2 and SET7/9, but negatively regulated by LSD1, which were enriched with genes involved in cell proliferation regulation. Importantly, YY2-regulated gene transcription, cell proliferation and tumor growth were dependent, at least partially, on YY2 K247 methylation. Finally, somatic mutations on YY2 found in cancer, which are in close proximity to K247, altered its methylation, DNA-binding activity and gene transcription it controls. Our findings revealed the first PTM with functional implications imposed on YY2 protein, and linked YY2 methylation with its biological functions.

## Introduction

Yin Yang 1 (YY1) is a ubiquitous multifunctional zinc-finger transcription factor that is involved in a variety of biological processes, including development, cell proliferation and differentiation, DNA repair, and apoptosis [1–7]. It is evolutionarily conserved throughout the vertebrate and invertebrate lineages. YY2, the paralog of YY1 in mouse and human, was initially identified through DNA and amino-acid sequence database analysis. Specifically, human YY2 shares 65 and 56% identity in the DNA and protein sequence, respectively, with YY1, and the most pronounced similarity found is in the zinc-finger regions of the two proteins [8]. Comparative genomic approaches revealed that *YY2* is a duplication product from *YY1* that has been generated through retroposition and inserted into another gene locus named *Mbtps2* (membrane-bound transcription factor protease site 2), which occurred after the divergence of placental mammals from other vertebrates based on the presence of *YY2* in only the placental mammals [9]. As opposed to YY1, the *YY2* gene is not ubiquitously expressed [8, 10]. Because of the high degree of conservation in the zinc-finger regions between YY1 and YY2, YY2 was shown to bind to the YY1-binding sequence 5′-(A/c/g)(A/ t)NATG(G/a/t)(C/a)(G/c/t)-3′*in vitro* as well as some YY1-bound gene promoters in cultured cells [8,11,12,13]. Similar to YY1, YY2 displays both transcriptional activation and repression functions [8]. Mouse embryonic fibroblast cells from mice carrying *yy1* alleles expressing various amounts of yy1 display a dosage-dependent requirement of yy1 for cell proliferation. Accordingly, inhibition of YY1 in cultured cells led to cytokinesis defects and cell cycle arrest [14]. In contrast, inhibition of YY2 resulted in accelerated cell proliferation and reversed the antiproliferative effects of YY1 deficiency [15]. Similarly, knockdown of YY1 or YY2 caused inverse changes in ultraviolet sensitivity, suggesting that YY2 is not redundant to YY1, and YY2 might have distinguished roles in cellular physiology [15]. The opposing functions of YY1 and YY2 could be due to that they compete for a common set of binding sites in the genome [8, 16], therefore regulating the transcription of a common set of genes in an opposite way [15]. Alternatively, YY1 and YY2 could possess distinct binding programs and, therefore, regulate unique gene sets. Chromatin immunoprecipitation coupled with sequencing (ChIP-seq) analysis revealed that YY1 binds in close proximity to the transcription start sites of many coding genes as well as intragenic and intergenic regions [17, 18]. However, the distribution of YY2 and its correlation with YY1 has never been assessed in a genome-wide scale.

A multitude of mechanisms have been shown to regulate the DNA-binding activity and function of YY1, such as its associated proteins, post-translational modifications (PTMs) and subcellular localization. YY1 has been shown to interact with several transcriptional factors, such as SP1, c-MYC, p53, GATA1 and GATA4, which can regulate YY1 function in transcription either in a cooperative or in a competitive manner [19–23]. In addition, YY1 was found to associate with an array of enzymes, which result in a variety of PTMs on YY1, such as poly(ADP-ribosyl)ation, ubiquitination, acetylation, O-linked glycosylation, S-nitrosation, sumoylation, phosphorylation and methylation. These PTMs can either regulate YY1-binding activity with DNA/proteins or YY1 protein stability, therefore regulating YY1 function in gene transcription, cell cycle and apoptosis control [24–37]. Recently, we reported that YY1 is targeted by SET7/9, and SET7/9-mediated lysine methylation of YY1 is critical for its DNA-binding activity [37]. YY1 was also known to be regulated by its subcellular distribution patterns, with its localization mainly being cytoplasmic at G1, nuclear at early and middle S and then cytosolic again in later S phase. Consequently, YY1 DNA-binding activity and the transcription of YY1-regulated replication-dependent histone genes increased markedly early in S phase [38]. YY1 transcriptional function was also shown to be repressed by cytoplasmic localization during *Xenopus laevis* development [39]. However, how YY2 DNA-binding activity and function is regulated, particularly by PTMs, remains completely unknown.

Histone methylation is a widespread type of chromatin modification that is known to influence chromatin structure and gene expression, therefore having important roles in biological processes in the context of development and cellular responses [40, 41]. Aberrant histone methylation has been linked with a variety of human diseases including cancers [42, 43]. It can occur on all basic residues: arginines, lysines and histidines [44, 45]. Arginine and lysine methylation have been extensively characterized on histones [46], which were dynamically regulated by a plethora of proteins called methyltransferases and demethylases, mediating the addition and removal of methyl groups from arginine and lysine residues on histones, respectively. It is now becoming clear that arginines and lysines in many non-histone proteins are also targeted for methylation and demethylation, which is functionally important in regulating the targeted proteins [47–49]. SET7/9, a lysine methyltransferase originally identified to modify histone H3 lysine 4, has been shown to target an array of non-histone proteins and to be implicated in a wide range of cellular functions [50–53]. For instance, p53 was the first non-histone protein reported to be targeted by SET7/9 for lysine methylation, which positively regulated p53 protein stability [54]. It was long thought that protein methylation was irreversible until lysine-specific demethylase 1 (LSD1), an amine oxidase, was found to specifically remove the methyl groups from mono- and di-methylated histone H3 lysine 4 by an oxidative demethylation reaction using flavin as cofactor [55]. Following the discovery of LSD1, the largest class of demethylase enzymes was identified, which contains a Jumonji C-domain and catalyze lysine demethylation of histones through an oxidative reaction that requires iron Fe (II) and α-ketoglutarate as cofactors [56]. Whereas LSD1 can only demethylate mono- and dimethyl histone H3 lysine 4/9, the Jumonji C-domain-containing histone demethylase family can remove all three forms of lysine methylation, mono-, di- and tri-methylation, and different members in this family targeted different methylated lysine or arginine residues in histones [57]. Similarly, histone demethylases can also target non-histone proteins and regulate the cellular functions of the targeted proteins [48].

Here we presented evidence that YY2 is methylated and demethylated at lysine 247 by SET7/9 and LSD1, respectively, and YY2 methylation regulates its affinity with the YY1 consensus-binding sequence *in vitro*. ChIP-seq analysis revealed that, similar as YY1, YY2 binds to gene promoter as well as intragenic and intergenic regions, with the most significantly enriched motif found to be the YY1 consensus-binding motif. Consistent with that YY2 methylation regulates its DNA-binding activity *in vitro*, SET7/9 and LSD1 regulate YY2 association to a subset of its binding sites in cultured cells. Consequently, SET7/9 and LSD1 exhibit opposing effects on the transcription of a subset of genes regulated by YY2 identified through RNA sequencing (RNA-seq), which were enriched with genes known to be involved in cell proliferation regulation. Furthermore, YY2’s inhibitory effects on cell proliferation and tumor growth were shown to be dependent on K247.

## Results

### YY2 is methylated by SET7/9 *in vitro*

**Figure 1:**
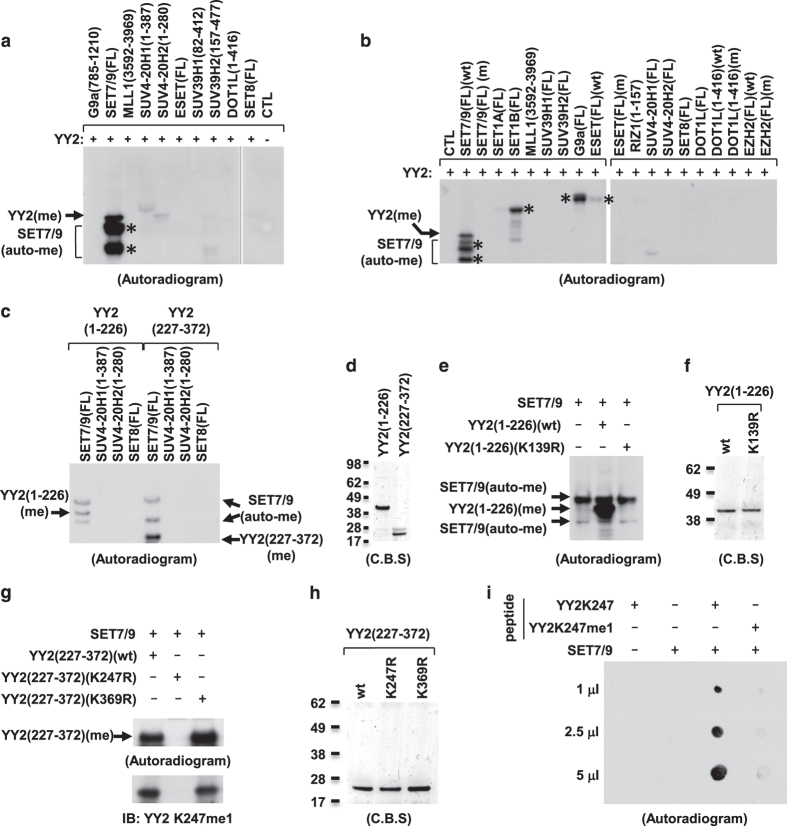
SET7/9 methylates YY2 *in vitro*. (**a**, **b**) *In vitro* methylation assay was performed by mixing purified bacterially expressed His-tagged YY2 protein with histone lysine methyltransferases (KMTs), either FL or truncations with enzymatic domain, from bacterial cells (**a**) or HEK293T cells with overexpression (**b**) as indicated, followed by autoradiogram. Stars indicate auto-methylation (auto-me) of KMTs. Black arrows indicate methylation of YY2 (YY2(me)). Wild type, wt; enzymatically dead mutant, m. (**c**) *In vitro* methylation assay was performed by mixing purified bacterially expressed His-tagged SET7/9 with YY2 amino- (1–226) or carboxyl terminus (227–372), followed by autoradiogram. (**d**) The expression of YY2 (1–226) and YY2 (227–372) in **c** was examined with the help of coomassie blue staining (C.B.S.). (**e**) *In vitro* methylation assay was performed by mixing purified bacterially expressed His-tagged SET7/9 with YY2 (1–226) wt or YY2 (1–226) with substitution of lysine 139 to arginine (K139R), followed by autoradiogram. (**f**) The expression of YY2 (1–226; wt) and YY2 (1–226; K139R) in **e** was examined by C.B.S. (**g**) *In vitro* methylation assay was performed by mixing purified bacterially expressed His-tagged SET7/9 with YY2 (227–372) wt or YY2 (227–372) with substitution of lysine 247 or 369 to arginine (K247R or K369R), followed by autoradiogram (top panel) or immunoblotting (IB) with anti-YY2K247me1 antibody (bottom panel). (**h**) The expression of YY2 (227–372; wt), YY2 (227–372; K247R) and YY2 (227–372; K369R) in **g** was examined by C.B.S. (**i**) *In vitro* methylation assay was performed by mixing synthetic short peptides from YY2 containing unmodified (K247) or monomethylated K247 (K247me1) with or without purified bacterially expressed SET7/9 proteins, followed by dot blot assay and autoradiogram as indicated.

Our recent work reported that YY1 was targeted for lysine methylation, which represented a new mechanism imposed on YY1 to regulate its DNA-binding activity and function [37]. To examine whether its paralog, YY2, is also subjected to lysine methylation, *in vitro* methylation assay was performed by mixing purified bacterially expressed YY2 with several histone lysine methyltransferases known to target to histone H3 or H4. It was found that YY2 was robustly methylated by SET7/9, and auto-methylation of SET7/9 was also observed (Figure 1a). To exclude the possibility that the observed YY2 methylation was contamination from SET7/9 enzyme preparation, a reaction with SET7/9 alone was included and served as a control, confirming YY2 methylation by SET7/9 (Supplementary Figure S1A). The expression and activity of all enzymes tested were shown in our recent work [37]. Of note, some of the enzymes tested displayed no activity when core histones were serving as substrates under current conditions [37]. To further test YY2 methylation by histone lysine methyltransferases, we included several additional enzymes, and purified all the enzymes from overexpressed HEK293T cells. It was found that wild-type (wt) SET7/9, but not its enzymatically dead mutant (m) or other methyltransferases tested, methylated YY2 (Figure 1b). In consistency with our recent work, SET7/9 as well as SET1B, G9a and ESET exhibited auto-methylation activity when purified from overexpressed HEK293T cells (Figure 1b). A control reaction with SET7/9 alone was performed to exclude the possibility that the observed YY2 methylation was contamination from enzyme preparation (Supplementary Figure S1B). The expression and activity of all enzymes tested were shown in our recent work [37]. We focused on studying YY2 methylation by SET7/9 in the current study, and meanwhile do not rule out the possibility that other enzymes might also be able to methylate YY2 under different experimental conditions.

Next, we sought to identify the lysine residues in YY2 targeted by SET7/9. Firstly, *in vitro* methylation assay was performed by incubating SET7/9 with YY2 truncations encompassing amino terminal (aa 1–226) or carboxyl terminal (aa227–372) of YY2 (Supplementary Figure S1C). It was found that both truncations were methylated by SET7/9, but not by other methyltransferases tested (Figure 1c). The expression of both truncations was determined by coomassie blue staining (C.B.S.; Figure 1d). It was reported previously that SET7/9 targets a consensus peptide sequence [K>R] [S>KYARTPN] K (in which the methylation site is underlined) for methylation [58, 59]. Examination of YY2 sequence revealed that there are three putative SET7/9 methylation sites, with one (K139) located at the amino- and two (K247 and K369) at carboxyl termini of YY2, respectively (Supplementary Figure S1C). Replacing lysine 139 with arginine (K139R) completely abolished SET7/9-mediated methylation of YY2 (1–226; Figure 1e). The expression of YY2 (1–226) and YY2 (1–226; K139R) was determined with C.B.S. (Figure 1f). Similarly, replacing lysine 247 with arginine (K247R) completely blocked SET7/9-mediated methylation of YY2 (227–372), whereas replacing another putative methylation site, lysine 369, with arginine (K369R) had no effects (Figure 1g, upper panel). YY2 (227–372), YY2 (227–372; K247R) and YY2 (227–372; K369R) were expressed equally well as determined with C.B.S. (Figure 1h). Methylation of YY2 K139 and K247 by SET7/9 was confirmed by using short peptides as substrates followed by matrix-assisted laser desorption/ionization time-of-flight mass spectrometry (MALDI-TOF MS) and LC-MS/MS analysis (Supplementary Figure S1D and E and Supplementary Table S1). We concluded so far that SET7/9 methylates YY2 at two lysine residues, K139 and K247, based on *in vitro* methylation assay.

To further demonstrate YY2 methylation by SET7/9 *in vitro*, YY2 protein after incubation with SET7/9 was subjected to mass spectrometry (MS) analysis. Two unique peptides containing monomethylated K247 were recovered, whereas no evidence was found to support the methylation of K139, which might be because of that the resultant peptide after tryptic cleavage was too short to be recovered (Supplementary Table S2 and Supplementary Figure S1C). To search for methylation of K139 and K247 in cultured cells, YY2 was purified from HeLa cells transfected with Flag-tagged YY2 and SET7/9, followed by MS analysis. It was found that K247 was monomethylated (me1), whereas no evidence was found to support K139 methylation under current conditions tested (Supplementary Table S3). We therefore focused on investigating YY2K247me1 and its function in the current study. Monomethylation of K247 by SET7/9 was further confirmed by using short peptides as substrates analyzed using dot blot assay (Figure 1i) and MALDI-TOF MS analysis (Supplementary Figure S1D). Specifically, peptide containing unmodified K247 was found to be methylated by SET7/9 robustly, whereas peptide containing monomethylated K247 (K247me1) no longer served as a substrate, suggesting SET7/9-mediated YY2 K247 monomethylation (Figure 1i and Supplementary Figure S1D). Importantly, K247 was found to be highly conserved during evolution, suggesting that methylation on this residue might be functionally important (Supplementary Figure S1F). It should be noted that YY2 K247 is homologous to YY1 K288, and differs from the two lysine residues, K173 and K411, in YY1 which we reported to be methylated by SET7/9 previously (Supplementary Figure S1G) [37].

### LSD1/AOF2 demethylates YY2 K247 monomethylation *in vitro*

**Figure 2:**
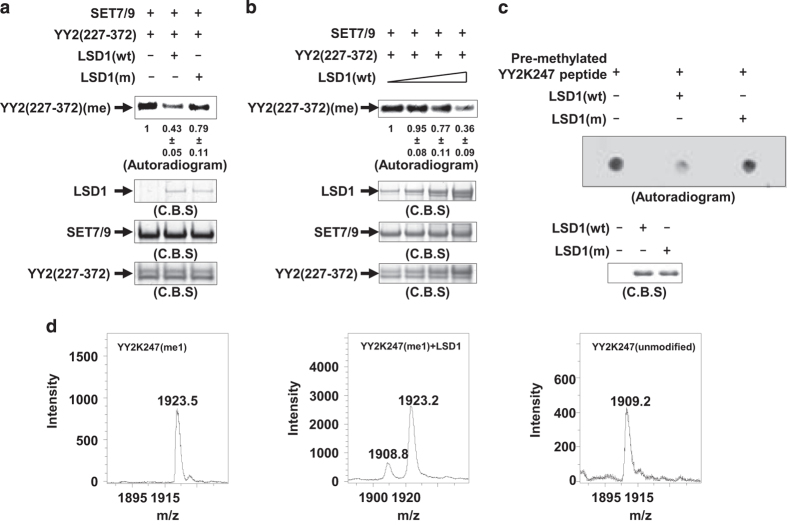
LSD1 demethylates YY2 K247 methylation *in vitro*. (**a**) YY2 (227–372) was pre-methylated by SET7/9, and then *in vitro* demethylation assay was performed by adding purified bacterially expressed His-tagged LSD1 wild type (wt) or its enzymatically dead mutant (m), followed by autoradiogram (top panel). Gel intensity was quantified by using Image J (National Institute of Health, Bethesda, MD, USA) and was shown as indicated. The expression of LSD1 (wt), LSD1 (m), SET7/9 and YY2 (227–372) was examined with C.B.S. (bottom panel). (**b**) *In vitro* demethylation assay was performed as described in **a** by adding increased amount of LSD1 (wt), followed by autoradiogram (top panel). Gel intensity was quantified by using Image J and was shown as indicated. The expression of LSD1 (wt), SET7/9 and YY2 (227–372) was examined with C.B.S. (bottom panel). (**c**) *In vitro* demethylation assay was performed by mixing SET7/9-pre-methylated K247 peptide and LSD1 (wt) or LSD1 (m), followed by dot blot assay and autoradiogram. The expression of LSD1 (wt) and LSD1 (m) was examined with C.B.S. (bottom panel). (**d**) *In vitro* demethylation assay was performed by mixing synthetic short peptides from YY2 containing monomethylated K247 (YY2K247me1) with or without purified bacterially expressed LSD1 protein, followed by MALDI-TOF MS analysis. YY2 K247 peptide (unmodified) served as a control.

LSD1, the first histone lysine demethylase discovered, has opposing activity to SET7/9 on histone as well as several non-histone proteins [55, 60, 61]. To test whether SET7/9-mediated YY2 K247 methylation is also targeted by LSD1, YY2 (227–372) was pre-methylated by SET7/9 followed by adding wt LSD1 or its enzymatically dead mutant (m), finding that LSD1 (wt) attenuated YY2 methylation and LSD1 (m) did so in a much less extent (Figure 2a, top panel). Loading of LSD1 (wt), LSD1 (m), SET7/9 and YY2 (227–372) was shown with C.B.S. (Figure 2a, bottom panels). Furthermore, LSD1 demethylated YY2 (227–372) in a dose-dependent manner (Figure 2b). Demethylation of K247me1 by LSD1 was confirmed by using short peptide containing K247, which was pre-methylated by SET7/9, as a substrate (Figure 2c). Finally, synthetic short peptide containing K247me1 was found to be demethylated by LSD1 analyzed using MALDI-TOF MS (Figure 2d).

### SET7/9 and LSD1 regulate YY2 K247 monomethylation in cultured cells

**Figure 3:**
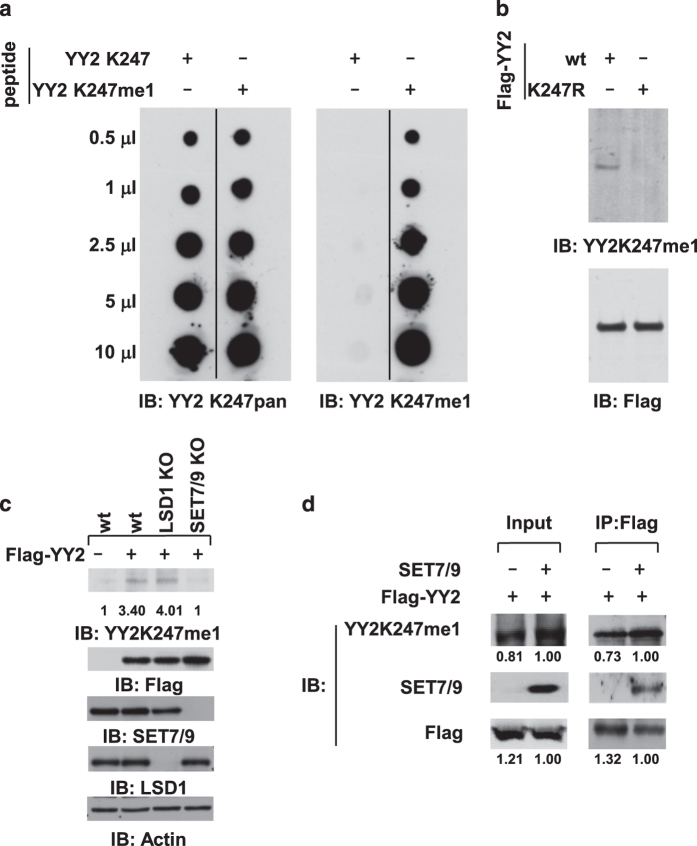
SET7/9 and LSD1 regulate YY2 K247 methylation in cultured cells. (**a**) Short peptides containing unmodified (YY2 K247) or monomethylated K247 (YY2K247me1) were prepared for dot blot assay, followed by IB using anti-YY2K247pan (left panel) or anti-YY2K247me1 (right panel) antibody as indicated. (**b**) HeLa cells transfected with vectors expressing Flag-tagged YY2 (wt) or YY2 (K247R) were subjected to IB with anti-YY2K247me1 or anti-Flag antibody as indicated. (**c**) Control (wt), SET7/9 or LSD1 KO HeLa cells transfected with control vector or vector expressing Flag-tagged YY2 were subjected to IB with antibodies as indicated. Intensity of YY2K247me1 was quantified by using Image J and normalized values were shown as indicated. (**d**) HeLa cells transfected with vector expressing Flag-tagged YY2 in the presence or absence of SET7/9 were subjected to IP with anti-Flag antibody followed by IB with antibodies as indicated. Intensity of YY2K247me1 was quantified by using Image J and normalized values were shown as indicated. Intensity of YY2K247me1 was quantified by using Image J and normalized values were shown as indicated.

To examine whether SET7/9 and LSD1 regulate YY2K247me1 in cultured cells, antibody detecting both unmethylated and monomethylated YY2 (anti-K247pan) or monomethylated lysine 247 only (anti-K247me1) was generated. To test the specificity of these antibodies *in vitro*, short peptides containing unmodified or monomethylated K247 were subjected to dot blotting with anti-K247pan or anti-K247me1 antibody. As expected, anti-K247pan antibody displayed similar affinity with both peptides, whereas anti-K247me1 antibody specifically recognized peptide containing monomethylated K247 (Figure 3a). To test the usage of anti-K247me1 antibody in cells, total lysates from HeLa cells transfected with Flag-tagged YY2 wt or mutant with substitution of lysine 247 to arginine (K247R) were subjected to immunoblotting (IB) with anti-K247me1 antibody. It was found that, despite both proteins expressed at a similar level (Figure 3b, bottom panel), anti-K247me1 antibody only detected YY2 (wt), but not YY2 (K247R), suggesting that it specifically recognizes YY2 protein (Figure 3b, upper panel). Of note, both anti-K247pan and anti-K247me1 antibodies failed to detect endogenous YY2 protein in the cell lines we tested, including HEK293T and HeLa cells.

Taking advantage of the anti-K247me1 antibody generated, we then tested whether SET7/9 and LSD1 regulate YY2 methylation in cultured cells by transfecting HeLa cells with control short interfering RNA (siRNA) or siRNA specifically targeting *SET7/9* or *LSD1* in the presence of Flag-tagged YY2 followed by IB with anti-K247me1 antibody. Surprisingly, knockdown of neither protein had a significant effect on YY2K247me1 levels globally (Supplementary Figure S2A), which could be because of that other enzymes yet to be discovered compensating the loss of SET7/9 and LSD1, or SET7/9 and LSD1, regulate YY2 methylation locally such as on chromatin. It is also known that, for some of the epigenetic enzymes, such as CARM1 [62], residual proteins after siRNA-mediated knockdown will still be fully functional in terms of its enzymatic activity. Therefore, CRISPR/Cas9-mediated gene editing was applied to generate SET7/9 and LSD1 knockout (KO) HeLa cell lines (Supplementary Figure S2B). SET7/9 KO cell line was described previously [37]. To our surprise, knocking out of SET7/9 nearly abolished, whereas LSD1 slightly increased K274 methylation (Figure 3c). This experiment suggested that SET7/9 was mainly, if not solely, responsible for K247 methylation in cultured cells. To further support that SET7/9 methylated YY2 in culture cells, overexpression of SET7/9 induced an increased level of YY K247me1, and it was found to interact with YY2 (Figure 3d).

### YY2 K247 regulates its DNA-binding activity

**Figure 4:**
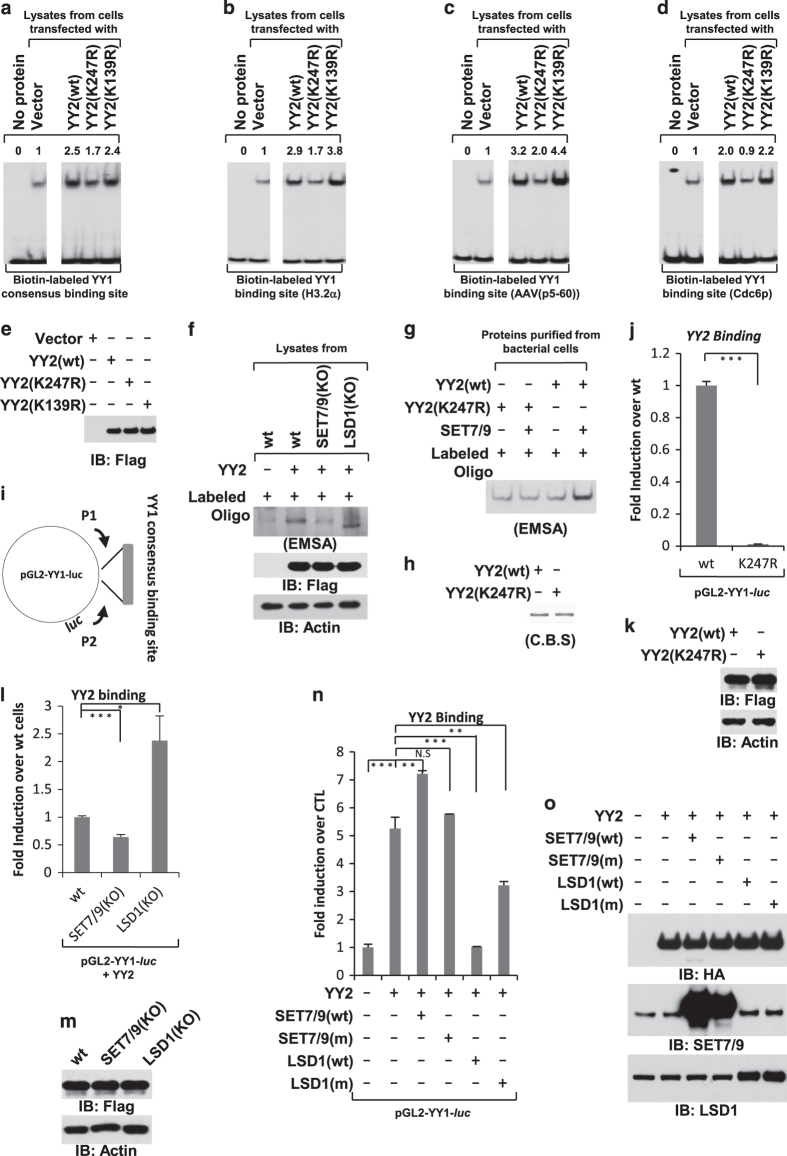
YY2 K247 methylation regulates YY2 DNA-binding activity. (**a**–**d**) DNA EMSA assay was performed by incubating biotinylated oligonucleotide containing YY1 consensus-binding site (**a**) or YY1-binding site in histone H3.2 gene-coding region (H3.2α; **b**), adeno-associated virus (AAV p5–60; **c**) or Cdc6 (Cdc6p) gene promoter region (**d**) with or without whole-cell lysates prepared from HeLa cells transfected with control vector or vectors expressing Flag-tagged YY2 (wt), YY2 (K247R) or YY2 (K139R). Intensity of shifted band was quantified by using Image J and was shown as indicated. (**e**) The expression of YY2 (wt), YY2 (K247R) and YY2 (K139R) in **a**–**d** was examined through IB with anti-Flag antibody. (**f**) DNA EMSA assay was performed by incubating biotinylated oligonucleotide containing YY1 consensus-binding site with whole-cell lysates prepared from control (wt), SET7/9 or LSD1 KO HeLa cells transfected with control vector or Flag-tagged YY2. (**g**) DNA EMSA assay was performed by incubating biotinylated oligonucleotide containing YY1 consensus-binding site with *in vitro*-purified bacterially expressed YY2 (wt) or YY2 (K247R) in the presence or absence of SET7/9. (**h**) The expression of YY2 (wt) and YY2 (K247R) in **g** was examined through C.B.S. (**i**) YY1 consensus-binding site was cloned into pGL2-luciferase vector (pGL2-YY1-*luc*). YY2 binding to the consensus-binding site can be examined through ChIP followed by qPCR using a primer set (P1 and P2) flanking the multiple cloning sites in pGL2 vector. (**j**) HeLa cells were co-transfected with pGL2-YY1-*luc* vector and vectors expressing HA-tagged YY2 (wt) or YY2 (K247R), followed by ChIP with anti-HA antibody and qPCR with primer set (P1+P2) as described in **i**. ChIP signals were presented as fold induction over wt after being normalized to input (±s.e.m., ***P<0.001). (**k**) The expression of YY2 (wt) and YY2 (K247R) in **j** was examined through IB. (**l**) Control (wt), SET7/9 or LSD1 KO HeLa cells were transfected with pGL2-YY1-*luc* vector in the presence of vector expressing HA-tagged YY2, followed by ChIP with anti-HA antibody and qPCR with primer set (P1+P2) as described in **g**. ChIP signals were presented as fold induction over wt cells after being normalized to input (±s.e.m., *P<0.05, ***P<0.001). (**m**) The expression of YY2 in **l** was examined through IB. (**n**) HeLa cells were transfected with pGL2-YY1-*luc* and control vector (CTL) or HA-tagged YY2 in the presence or absence of SET7/9 (wt), SET7/9 (m), LSD1 (wt) or LSD1 (m), followed by ChIP with anti-HA antibody and qPCR with primer set (P1+P2) as described in **I**. ChIP signals were presented as fold induction over CTL after being normalized to input (±s.e.m., **P<0.01, ***P<0.001, NS, nonsignificant). (**o**) The expression of YY2, SET7/9 (wt), SET7/9 (m), LSD1 (wt) and LSD1 (m) as described in **n** was examined by IB using antibodies as indicated.

Both YY1 and YY2 are believed to exert their biological functions primarily based on their DNA-binding activity. It was reported previously that YY2 binds to the YY1 consensus-binding sequence (5′-(A/c/g)(A/t)NATG(G/a/t)(C/a)(G/c/t)-3′) with a similar affinity as YY1 *in vitro* [8, 11, 13]. To examine whether SET7/9-mediated YY2 K247 methylation regulates its DNA-binding activity, we first examined whether K247 is critical. Electrophoretic mobility shift assay (EMSA) was performed by mixing biotinylated oligonucleotide containing the YY1 consensus-binding site with whole-cell extracts collected from HeLa cells transfected with a control vector or vectors expressing YY2 (wt), YY2 (K139R) or YY2 (K247R). The specificity of the biotinylated oligonucleotide containing YY1 consensus-binding site was shown previously [37]. It was found that incubation with extracts from control vector-transfected cells led to a weak shift of the biotinylated oligonucleotide, which could be the result of endogenous YY1 and/or YY2 binding (Figure 4a, compared lane 1 to lane 2). As expected, incubation with extracts from YY2 (wt)-transfected cells led to a much stronger shift (Figure 4a, compared lane 3 to lane 2). Interestingly, YY2 (K247R) attenuated (less shift; Figure 4a, compared lane 4 to lane 3), whereas YY2 (K139R) had no significant impact on YY2 DNA-binding activity (Figure 4a, compared lane 5 to lane 3). To further demonstrate that K247 is involved in YY2 binding with DNA, EMSA was performed with three additional biotinylated oligonucleotides containing the YY1-binding site in histone H3.2 gene-coding region (H3.2α), adeno-associated virus or Cdc6 gene promoter region (AAV p5–60 or Cdc6p). Similarly, YY2 (K247R) was found to bind to all three oligonucleotides more weakly compared with YY2 (wt) and YY2 (K139R; Figure 4b–d). Of note, YY2 (wt), YY2 (K247R) and YY2 (K139R) were expressed equally well as assessed by IB (Figure 4e). To further demonstrate that K247 methylation is involved in YY2 binding with DNA, EMSA was performed with extracts from wt, SET7/9 or LSD1 KO HeLa cells transfected with YY2. It was found that SET7/9 KO led to a marked decrease in YY2 binding with the YY1 consensus-binding site, whereas LSD1 KO reproducibly resulted in a faster migration, indicating that the composition of YY2 complex was altered, which might potentially be due to that YY2 methylation was changed (Figure 4f, upper panel). Expression of YY2 in these cells was shown (Figure 4f, bottom two panels). Finally, EMSA was performed by mixing *in vitro-*purified bacterially expressed His-tagged YY2 (wt) or YY2 (K247R) with SET7/9 protein. It was found that both bacterially expressed YY2 (wt) and YY2 (K247R) bound with YY1 consensus site with similar affinity in the absence of SET7/9, indicating that the decreased binding of K247R with DNA observed when using cell lysates was not simply due to that mutation of K247 altered YY2 conformation (Figure 4g, compared lane 1 to lane 3). More importantly, YY2 (wt), but not YY2 (K247R), binding with DNA was enhanced in the presence of SET7/9, suggesting that SET7/9-mediated YY2 K247 methylation was involved in YY2 binding with DNA (Figure 4g, compared lane 2 to lane 4). The expression of YY2 (wt) and YY2 (K247R) was determined by C.B.S. (Figure 4h).

We next sought to examine whether YY2 binding with DNA is regulated by SET7/9-mediated methylation in cultured cells. Firstly, we examined whether SET7/9 or mutation of K247 will alter YY2 subcellular localization. Wt or SET7/9 (KO) HeLa cells were transfected with Flag-tagged YY2 followed by immunofluorescence (IF) analysis, finding that YY2 protein retained in the nucleus in both cell lines (Supplementary Figure S3A). Furthermore, overexpression of SET7/9 had no significant impact on the subcellular localization of YY2 either (Supplementary Figure S3B). We also transfected HeLa cells with YY2 (wt) or YY2 (K247R) followed by IF analysis and found no significant difference on the cellular localization of these two proteins (Supplementary Figure S3C). We therefore went forward to examine whether SET7/9-mediated K247 methylation regulates YY2 DNA-binding activity. HeLa cells were transfected with luciferase vector containing the YY1 consensus-binding site (pGL2-YY1-*luc*), which we have described previously [37], and vectors expressing HA-tagged YY2 (wt) or YY2 (K247R), followed by ChIP assay with anti-HA antibody. Binding of YY2 was examined through quantitative polymerase chain reaction (qPCR) by using primer set specifically targeting to the luciferase vector encompassing the YY1-binding site (Figure 4i). Consistent with our EMSA data, YY2 (K247R) displayed weaker binding to the consensus site compared with YY2 (wt; Figure 4j and k). To further examine whether YY2 binding with DNA is regulated by SET7/9-mediated methylation in cultured cells, control (wt), SET7/9 and LSD1 KO cells were transfected with pGL2-YY1-*luc* and HA-tagged YY2 followed by ChIP with anti-HA antibody. Consistent with the observation from the experiment shown in Figure 3c, SET7/9 KO led to a decreased, whereas LSD1 KO resulted in an increased binding of YY2 with YY1-binding site (Figure 4l and m). Finally, HeLa cells were transfected with pGL2-YY1-*luc* and HA-tagged YY2 in the presence or absence of SET7/9 (wt), SET7/9 (m), LSD1 (wt) or LSD1 (m), followed by ChIP assay with anti-HA antibody. It was found that co-transfection of SET7/9 (wt) resulted in a further increase, whereas SET7/9 (m) caused no significant change of YY2 binding; co-transfection of LSD1 (wt) led to a decreased YY2 binding to the YY1-binding site, whereas LSD1 (m) displayed a weaker effect compared with LSD1 (wt; Figure 4n). The expression of YY2, SET7/9 (wt), SET7/9 (m), LSD1 (wt) and LSD1 (m) was examined by IB (Figure 4o). Taken together, our data *in vitro* and in cultured cells suggested that YY2 K247 regulates its DNA-binding affinity with the YY1 consensus-binding sequence, and is consistent with the observation that SET7/9 and LSD1 regulate YY2 methylation.

### YY2 K247 monomethylation regulates its genomic association

**Figure 5:**
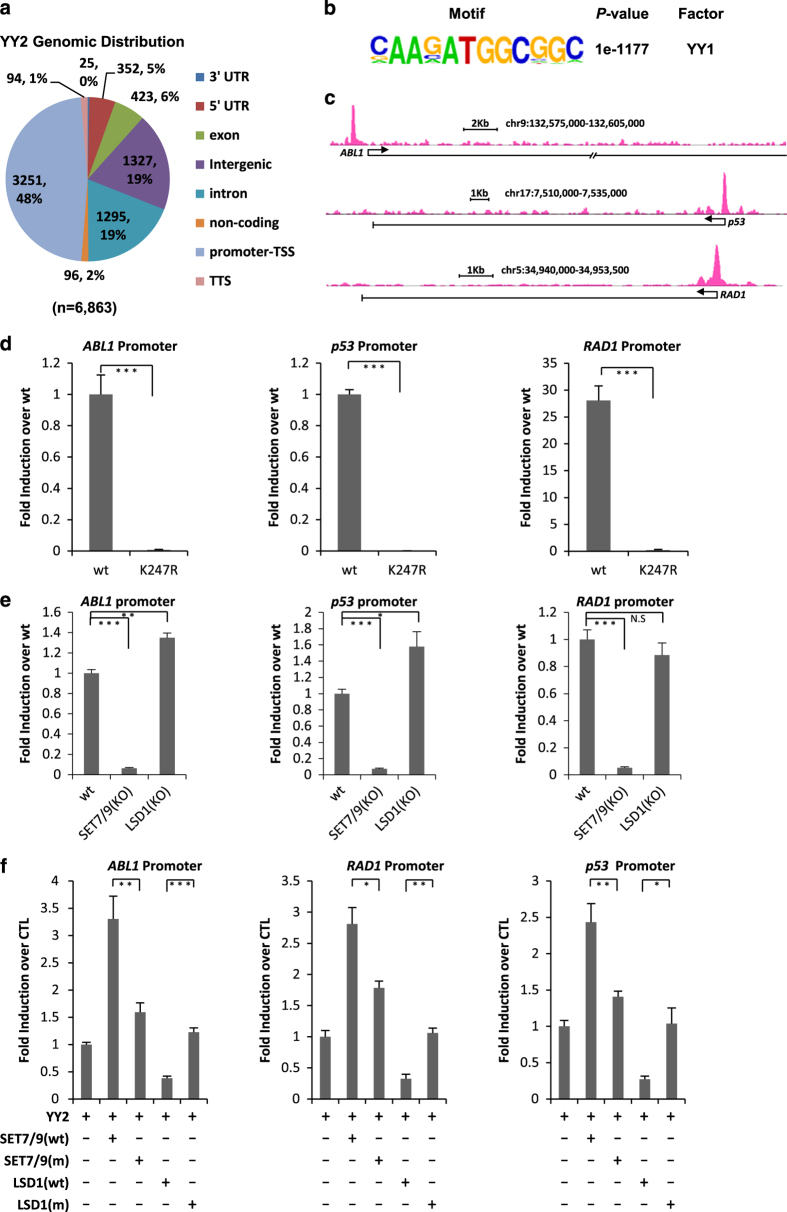
YY2 K247 methylation regulates YY2 binding with chromatin. (**a**, **b**) Inducible HeLa cells expressing pRevTRE-Flag-HA-YY2 were treated with doxycycline (Dox, 600 μgml^−1^) for 48 h, followed by ChIP-seq to detect YY2-binding sites in the genome. Peak finding (**a**) and motif analysis (**b**) were performed using HOMER. (**c**) YY2 binding detected by ChIP-seq was shown for *ABL1*, *TP53/p53* and *RAD1* genes as indicated. (**d**) HeLa cells were transfected with vectors expressing HA-tagged YY2 (wt) or YY2 (K247R), followed by ChIP with anti-HA antibody and qPCR with primers specifically targeting promoter regions of selected genes as indicated. ChIP signals were presented as fold induction over wt after being normalized to input (±s.e.m., ***P<0.001). (**e**) Control (wt), SET7/9 or LSD1 KO HeLa cells were transfected with vector expressing HA-tagged YY2, followed by ChIP with anti-HA antibody and qPCR with primer specifically targeting promoter regions of selected genes as indicated. ChIP signals were presented as fold induction over wt after being normalized to input (±s.e.m., *P<0.05, **P<0.01, ***P<0.001, NS, nonsignificant). (**f**) HeLa cells were transfected with vectors expressing HA-tagged YY2 in the presence or absence of SET7/9 (wt), SET7/9 (m), LSD1 (wt) or LSD1 (m), followed by ChIP with anti-HA antibody and qPCR with primer specifically targeting promoter regions of selected genes as indicated. ChIP signals were presented as fold induction over CTL (YY2 alone) after being normalized to input (±s.e.m., *P<0.05, **P<0.01, ***P<0.001).

YY2 was shown to bind to some gene promoters known to be bound by YY1 in cultured cells [8]. However, the genome-wide distribution of YY2 protein on chromatin in somatic cells remains unknown. Despite numerous attempts, our ChIP-seq with anti-YY2 antibodies was not successful. Taking advantage of the HA-tagging system, which has been proven to be effective in ChIP-seq, we generated HeLa cell line inducibly expressing HA-tagged YY2 and performed ChIP-seq to examine YY2 binding on chromatin. We found that around 48% of YY2-binding sites located on gene promoter regions (transcription start site), whereas the rest 52% were found on 3′ untranslated region (UTR), 5′ UTR, exon, intron, transcription termination site, intergenic or non-coding RNA regions (Figure 5a). Motif analysis revealed that the most enriched DNA motif in all YY2-binding sites was indeed the YY1 consensus-binding motif, which suggested that the YY2 ChIP-seq was valid (Figure 5b). YY2 binding detected by ChIP-seq was shown on some selected gene promoters, such as *ABL1*, *TP53/p53* and *RAD1* (Figure 5c).

To test whether SET7/9-mediated methylation at K247 regulates YY2 binding on endogenous gene loci, HeLa cells were transfected with vectors expressing HA-tagged YY2 (wt) or YY2 (K247R) followed by ChIP assay with anti-HA antibody. It was found that binding of YY2 (K247R) on selected gene promoters, including *ABL1*, *p53*, *RAD1*, *CCNT2* and *CCNA2*, was significantly lower compared with YY2 (wt; Figure 5d, Supplementary Figure S4A and B). Furthermore, control (wt), SET7/9 and LSD1 KO cells were transfected with HA-tagged YY2 followed by ChIP with anti-HA antibody. Consistent with our observation that KO of SET7/9 and LSD1 resulted in a decreased and an increased levels of K247me1, respectively, YY2 binding was affected in an opposite direction in SET7/9 and LSD1 KO cells on selected gene promoters except that LSD1 KO had no significant impact on YY2 binding with *RAD1* promoter (Figure 5e, Supplementary Figure S4C and D). Finally, HeLa cells were transfected with HA-tagged YY2 and vectors expressing SET7/9 (wt), SET7/9 (m), LSD1 (wt) or LSD1 (m), followed by ChIP assay with anti-HA antibody. Again, co-transfection of SET7/9 (wt) led to a much more significant increase in YY2 binding compared with SET7/9 (m). Meanwhile, co-transfection of LSD1 (wt) resulted in a much more significant decrease in YY2 binding compared with LSD1 (m) on selected gene promoters, such as *ABL1*, *RAD1* and *p53*, supporting the notion that YY2 methylation regulates its binding with DNA (Figure 5f and Supplementary Figure S4E). Taken together, our data suggested that YY2 K247 regulates its binding affinity with endogenous genomic loci, and was consistent with the observation that SET7/9 and LSD1 regulate YY2 methylation.

### YY2 K247 monomethylation is involved in YY2-regulated gene transcriptional program

**Figure 6:**
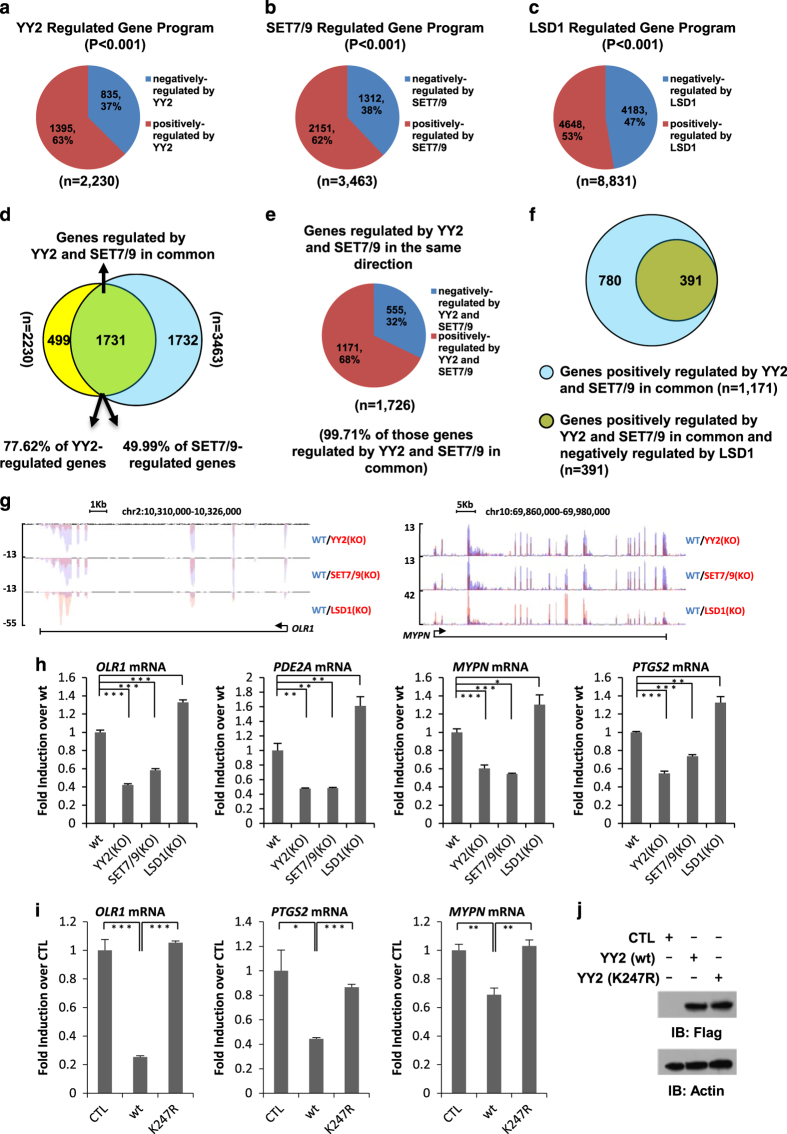
YY2 K247 methylation is involved in YY2-regulated gene transcriptional program. (**a**–**c**) RNA prepared from control (wt), YY2, SET7/9 or LSD1 KO HeLa cells were subjected to RNA-seq analysis. Pie chart was used to display genes positively or negatively regulated by YY2 (**a**), SET7/9 (**b**) or LSD1 (**c**; *P*<0.001). (**d**) Venn diagram showing overlapping between YY2 and SET7/9-regulated gene programs. (**e**) Pie chart showing gene programs regulated by YY2 and SET7/9 in the same direction including positively and negatively regulated genes (*P*<0.001). (**f**) Venn diagram showing overlapping between gene programs negatively regulated by LSD1 and those positively regulated by YY2 and SET7/9 in common. (**g**) Genome browser view of RNA-seq as described in **a**–**c** for selected genes as indicated was shown. (**h**) RNA prepared from control (wt), YY2, SET7/9 or LSD1 KO HeLa cells was subjected to RT-qPCR analysis to examine mRNA levels of genes as indicated. Data shown were the relative fold change compared with control samples after normalization to actin (±s.e.m., *P<0.05, **P<0.01, ***P<0.001). (**i**) HeLa cells were transfected with control vector or vectors expressing YY2 (wt) or YY2 (K247R), followed by RT-qPCR analysis to examine mRNA levels of selected genes as indicated. Data shown were the relative fold change compared with control samples after normalization to actin (±s.e.m., *P<0.05, **P<0.01, ***P<0.001). (**j**) HeLa cells as described in **i** were subjected to IB analysis using antibodies as indicated.

Similar to YY1, YY2 can function as a gene transcriptional regulator, which is primarily based on its DNA-binding activity [8]. Our observation that SET7/9-mediated YY2 methylation regulates YY2-binding affinity with DNA prompted us to examine whether it is involved in YY2-regulated gene transcriptional program. To identify gene programs regulated by YY2, SET7/9 or LSD1, we generated YY2 (Supplementary Figure S5A), SET7/9 [37] or LSD1 (Supplementary Figure S2B) KO cells by using CRISPR/Cas9-mediated gene-editing technology followed by RNA-seq analysis. It was found that all three proteins regulated a large set of genes, with YY2, SET7/9 and LSD1 regulating 2 230, 3 463 and 8 831 genes, respectively (Figure 6a–c). The top-enriched gene ontology (GO) terms for genes positively or negatively regulated by YY2, SET7/9 or LSD1 were shown (Supplementary Table S4). Particularly, consistent with YY2 function in cell cycle/cell proliferation regulation, such GO term was indeed found to be enriched in YY2 positively regulated genes. Intriguingly, cell cycle/cell proliferation was also found to be enriched in SET7/9 positively regulated genes, but in LSD1 negatively regulated genes (Supplementary Table S3). Comparing YY2-regulated genes with those of SET7/9, we found that 1 731 genes were regulated by both proteins in common, which was around 78 and 50% of genes regulated by YY2 and SET7/9, respectively (Figure 6d). More importantly, nearly all of these 1 731 genes (~99.71%) were regulated by both proteins in the same direction, with 68 and 32% of them being positively and negatively regulated in common by YY2 and SET7/9, respectively (Figure 6e). In accordance with their functional roles in cell cycle/cell proliferation regulation, GO analysis of genes positively regulated by YY2 and SET7/9 in common revealed that cell cycle/cell proliferation was among the top most enriched terms (Supplementary Table S4). Interestingly, around one-third of genes positively regulated by YY2 and SET7/9 in common were found to be negatively regulated by LSD1, and cell cycle/cell proliferation, again, was found to be among the top most enriched terms, suggesting that YY2 methylation might be involved in the regulation of expression of these genes and possibly cell cycle/cell proliferation function (Figure 6f and Supplementary Table S4). UCSC genome browser track views for selected genes were shown as indicated (Figure 6g), which were further validated by reverse transcriptase qPCR (RT-qPCR) analysis (Figure 6h).

To test whether SET7/9-mediated YY2 K247 methylation is involved in YY2-regulated gene transcription, HeLa cells were transfected with control vector or vectors expressing YY2 (wt) or YY2 (K247R), followed by RT-qPCR analysis to examine the expression of several genes mentioned above. Unexpectedly, instead of further activating them, overexpression of YY2 resulted in either decreased or unaltered expression of these genes (Figure 6i and data not shown), which was consistent with previous reports that YY2 as well as its human homolog, YY1, could be switched from an activator to repressor when present at high levels [8, 37]. Nevertheless, YY2’s repressive effects on these genes appeared to be attenuated when K247 was mutated (Figure 6i). Both YY2 (wt) and YY2 (K247R) were expressed equally well as determined by IB (Figure 6j). To further demonstrate YY2’s repressive effects on gene transcription when present at high levels were dependent on K247, HeLa cells were transfected with luciferase reporter vector containing YY1 consensus-binding site (pGL2-YY1-*luc*), and vectors expressing YY2 (wt) or YY2 (K247R), followed by luciferase reporter activity measurement. Consistently, overexpression of YY2 (wt) led to a significant decrease in luciferase reporter activity, whereas YY2 (K247R) lost its repressive effect (Supplementary Figure S5B and S5C). Taken together, our data suggested that YY2 K247 and the enzymes, both SET7/9 and LSD1, regulating its methylation are involved in the transcription, at least some, of those YY2-regulated genes.

### YY2 somatic mutations found in cancer altered K247 monomethylation, DNA-binding activity and its regulated gene transcription

**Figure 7:**
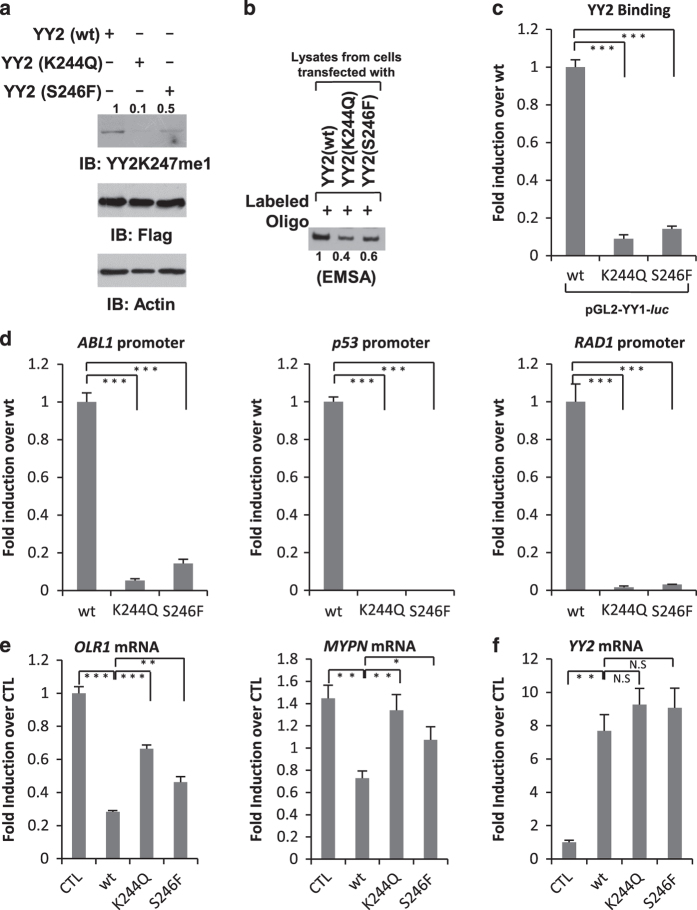
YY2 somatic mutations alter K247 methylation, DNA-binding activity and its regulated gene transcription. (**a**) HeLa cells transfected with vector expressing Flag-tagged YY2 (wt), YY2 (K244Q) or YY2 (S246F) were subjected to IB with antibodies as indicated. Intensity of YY2K247me1 was quantified using Image J and is shown as indicated. (**b**) DNA EMSA assay was performed as described in Figure 4a with whole-cell lysates prepared from HeLa cells transfected with vector expressing Flag-tagged YY2 (wt), YY2 (K244Q) or YY2 (S246F). Intensity of shifted band was quantified by using Image J and shown as indicated. (**c**) HeLa cells were transfected with pGL2-YY1-*luc* vector and vectors expressing HA-tagged YY2 (wt), YY2 (K244Q) or YY2 (S246F), followed by ChIP and qPCR as described in Figure 4h. ChIP signals were presented as fold induction over wt after being normalized to input (±s.e.m., ***P<0.001). (**d**) HeLa cells were transfected with vectors expressing HA-tagged YY2 (wt), YY2 (K244Q) or YY2 (S246F), followed by ChIP as described in Figure 5d. ChIP signals were presented as fold induction over wt after being normalized to input (±s.e.m., ***P<0.001). (**e**, **f**) HeLa cells were transfected with control vector or vectors expressing YY2 (wt), YY2 (K244Q) or YY2 (S246F), followed by RT-qPCR analysis to examine mRNA levels of selected genes as indicated. Data shown were the relative fold change compared with control samples after normalization to actin (±s.e.m., *P<0.05, **P<0.01, ***P<0.001; NS, nonsignificant).

To further explore the functional significance of YY2 methylation, we sought to examine the impact of two somatic mutations in close proximity to K247, K244Q and S246F (lysine 244 to glutamine and serine 246 to phenylalanine), on K247 methylation, DNA-binding activity and its regulated gene transcription. K244Q and S246F mutations were identified in colon and skin cancer, respectively, in COSMIC database. Firstly, HeLa cells transfected with Flag-tagged YY2 (wt), K244Q or S246F) were subjected to IB with antibodies as indicated (Figure 7a). It was found that, despite all three proteins expressed at a similar level (Figure 7a, middle panel), K244Q and S246F significantly attenuated YY2 K247 methylation (Figure 7a, upper panel). Consistent with our observation that YY2 K247 methylation regulates its DNA-binding activity, both K244Q and S246F expressed in HeLa cells exhibited a decreased binding affinity to YY1 consensus-binding site compared with YY2 (wt) as demonstrated by EMSA (Figure 7b) and ChIP assay (Figure 7c and Supplementary Figure S6A). It should be noted that K244Q also affected YY2 DNA-binding activity when purified from bacterial cells, indicating that K244 itself was involved in YY2 conformation and the resultant decrease of K247me1 as described in Figure 7a, for the most, might only partially account for the decreased binding affinity with DNA of K244Q in cultured cells (Supplementary Figure S6B). Furthermore, to test whether K244Q and S246F alter YY2 binding on endogenous gene loci, HeLa cells were transfected with vectors expressing HA-tagged YY2 (wt), YY2 (K244Q) or YY2 (S246F) followed by ChIP assay with anti-HA antibody. It was found that binding of YY2 (K244Q) and YY2 (S246F) on selected gene promoters, including *ABL1*, *p53*, *RAD1*, *CCNT2* and *CCNA2*, was significantly lower compared with YY2 (wt; Figure 7d, Supplementary Figure S6C and S6D). Finally, YY2’s repressive effects on gene transcription on selected genes, including *OLR1* and *MYPN*, were attenuated for K244Q and S246F (Figure 7e). The expression of YY2 (wt), YY2 (K244Q) and YY2 (S246F) was expressed at a similar level (Figure 7f and Supplementary Figure S6E). Taken together, our data demonstrated that YY2 somatic mutations found in cancer, such as K244Q and S246F, could alter K247 methylation, DNA-binding activity and its regulated gene transcription, suggesting that K247 methylation could be pathologically relevant.

### YY2 K247 monomethylation is involved in YY2-regulated cell proliferation and tumor growth

**Figure 8:**
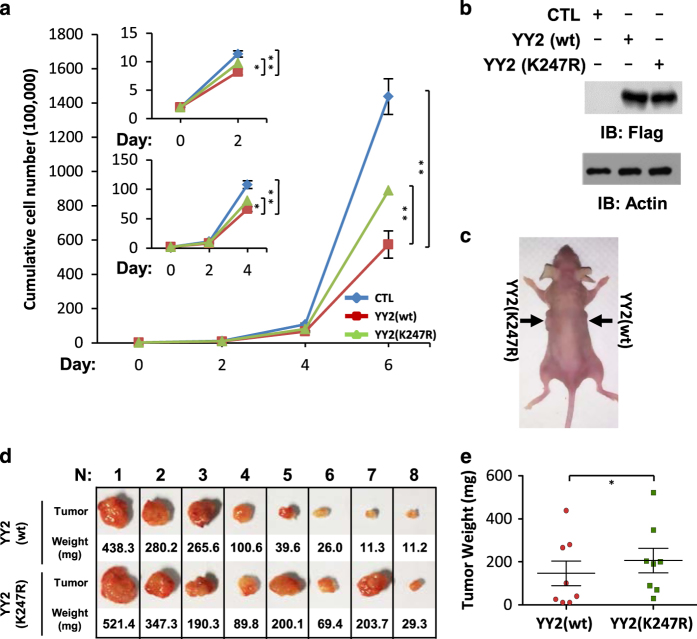
YY2 K247 methylation is involved in YY2-regulated cell proliferation and tumor growth. (**a**) HeLa cells were infected with control lentiviral vector or vectors expressing YY2 (wt) or YY2 (K247R), followed by cell number counting every 2 days. Significance test for the change of cell number between CTL and YY2 (wt), or YY (wt) and YY2 (K247R) on days 2 (inset), 4 (inset) and 6 were shown as indicated (±s.e.m., *P<0.05, **P<0.01). (**b**) HeLa cells as described in **a** were subjected to IB analysis using antibodies as indicated. (**c**) HeLa cells as described in **a** were injected into female athymic Nu/Nu mice as shown. (**d**) Mice as described in **c** were killed 3 weeks after subcutaneous injection, and tumors were then excised, photographed and weighed. (**e**) Significance test for the weight of tumors shown in **d** was performed.

It has been reported previously that YY2 displays an inhibitory effect on cell proliferation [15]. Our findings that YY2 K247 and possibly its methylation are involved in the transcriptional regulation of some of those YY2-regulated genes, a subset of which has been shown to be implicated in cell proliferation, prompted us to examine whether YY2 K247 is involved in its regulated cell proliferation. The proliferation rate of HeLa cells expressing a control lentiviral vector or vectors encoding YY2 (wt) or YY2 (K247R) was recorded. In accordance with its role in inhibiting cell proliferation, cells expressing YY2 (wt) grew much slower compared with control cells. Importantly, YY2 (K247R) attenuated YY2 inhibitory effects on cell proliferation (Figure 8a). Both YY2 (wt) and YY2 (K247R) were expressed equally well as determined by IB (Figure 8b). K247R has an impact on YY2 inhibitory effect on cell proliferation was confirmed by colony formation assay (Supplementary Figure S7A and S7B). Furthermore, we tested whether YY2 K247 is involved in YY2-mediated tumor growth. Nude mice were implanted with HeLa cells infected with lentiviral vectors encoding YY2 (wt) or YY2 (K247R) on right or left side of the body, respectively (Figure 8c). Tumors were collected 3 weeks after implantation, and it was found that weight and size of tumors from HeLa cells infected with YY2 (K247R) were in general heavier and larger, respectively, than YY2 (wt; Figure 8d). Significance test for tumor weight was performed as shown (Figure 8e). Taken together, our data suggested that K247 in YY2 is involved in YY2-regulated cell proliferation and tumor growth.

## Discussion

YY1 is a ubiquitous and multifunctional zinc-finger transcription factor that is involved in a variety of biological processes [1–7]. A multitude of mechanisms have been shown to regulate the DNA-binding activity and function of YY1 [19–39]. YY2, the human paralog of YY1, was shown to possess DNA-binding activity, and was proposed to have important biological roles, including gene transcriptional control, cell cycle regulation and ultraviolet damage response [15]. Despite the awareness of its importance in cellular physiology, how YY2 activity is regulated, particularly by post-translational modifications, and the functional consequence of such regulation remain completely unknown. In the present study, we provided evidence that YY2 was methylated by SET7/9 at a highly conserved lysine residue, K247, which is located right in front of the first zinc finger of YY2, suggesting a potential function of this modification in regulating YY2 DNA-binding activity. Indeed, YY2 K247 was found to regulate YY2-binding activity with the YY1 consensus-binding sequence determined with EMSA. To assess whether YY2 binding with chromatin in cultured cells is regulated by K247 methylation, its binding sites in the genome were revealed through ChIP-seq analysis. Furthermore, YY2 K247 and the enzymes involved in K247 methylation, SET7/9 and LSD1, were shown to be involved in YY2 binding with selected genomic loci. RNA-seq analysis in HeLa cells revealed that YY2 regulated the expression of a subset of genes with implications in cell proliferation. Importantly, SET7/9-mediated YY2 methylation was shown to involve in the transcriptional activation of some of these selected genes. Consequently, YY2 inhibitory effect on cell proliferation and tumor growth was dependent on YY2 K247, revealing an intimate link between YY2 methylation and its physiological functions. Significantly, YY2 somatic mutations found in cancer were shown to alter K247 methylation, DNA-binding activity and its regulated gene transcription, suggesting a potential role of YY2 K247 methylation in pathological conditions.

Initially, we sought to examine whether YY2 is similarly targeted for lysine methylation as YY1 through screening a panel of histone lysine methyltransferases, finding that YY2 was robustly methylated by SET7/9 in *vitro* (Figure 1a and b). Mutagenesis combined with MS analysis revealed that lysine 247, which is in close proximity to the first zinc finger in YY2, was monomethylated (me1) by SET7/9 (Figure 1c–i, Supplementary Figure S1A–E and Supplementary Tables S2 and S3). As expected, LSD1, the demethylase with opposing activity to SET7/9 on histone as well as several non-histone proteins was found to demethylate YY2K247me1 (Figure 2a–d). To the best of our knowledge, K247me1 described in the current study is the first PTM with functional characterization found on YY2. On the basis of our MS analysis, similar as YY1, YY2 is subjected to various types of PTMs on multiple sites, including phosphorylation, acetylation and methylation (unpublished data). Therefore, one can envision that, similar as YY1, YY2 activity and function will be subjected to the regulation of these various PTMs.

The high degree of evolutionary conservation of K247 in YY2 protein suggested that methylation on this site could be functionally important (Supplementary Figure S1F). Because of its close proximity to the first zinc finger, we reasoned that methylation of K247 might affect YY2 DNA-binding activity. Indeed, YY2 K247 methylation was involved in its binding with the YY1 consensus-binding sites *in vitro* and with chromatin in cultured cells (Figures 4 and 5 and Supplementary Figure S4). One interesting feature of YY2 localization in the genome as revealed by ChIP-seq was that, despite YY2 was found to be largely localized on promoter regions, majority of YY2-regulated genes exhibited no YY2 binding on their own promoter regions, suggesting that they might be regulated in a distal manner, which certainly remains as a great topic for future investigation.

The functional importance of YY2 K247 methylation was further supported by our observation that K247 was involved in its regulated gene transcription and inhibitory effects on cell proliferation and tumor growth (Figure 6,Figure 8). In addition, YY2 somatic mutations found in cancer altered K247 methylation, its DNA-binding activity and its regulated gene transcription (Figure 7). YY1 has been suggested to have an essential role in multiple types of cancers, which was mainly due to its elevated expression levels in cancers and its functional role in promoting cell proliferation. Therefore, it will be valuable to investigate whether YY2 expression and hence K247 methylation described in the present study are also dynamically regulated in cancers.

## Materials and methods

### Plasmids and cloning procedures

YY2 full-length or truncations were PCR-amplified from cDNA samples prepared from HEK293T cells by using KOD Hot Start DNA Polymerase (Novagen, Madison, WI, USA) and then cloned into p3XFLAG-CMV-10 (Sigma, St Louis, MO, USA), pET-28a (+) (Novagen); pRevTRE (Clontech, Palo Alto, CA, USA) or pBobi expression vector. Flag- and HA-tag was added to the amino- and carboxy termini of YY2, respectively, when cloning pRevTRE- and pBobi-YY2. LSD1 was PCR-amplified from cDNA samples prepared from HEK293T cells and then cloned into pCDNA3 (Invitrogen, Carlsbad, CA, USA) or pET-28a (+) (Novagen) expression vector. Cloning of all histone methyltransferases was described previously [37]. All point mutations were generated by using QuikChange Lightning Site-Directed Mutagenesis Kit (Stratagene, La Jolla, CA, USA). Luciferase reporter constructs containing YY1 consensus-binding motif ( 5′-CGCTCCCCGGCCATCTTGGCGGCTGGT-3′) or its mutant form (5′- CGCTCCGCGATTATCTTGGCGGCTGGT-3′) were described previously [37].

### Short interfering RNAs, antibodies and peptides

siRNA specifically targeting *YY2* ( 5′-CAGCTGGCAGAATTTACTAAA-3′), *SET7/9* (5′- TAGGGCCAGGGTATTATTATA-3′) or *LSD1/AOF2* (3′- CTGGAAATGACTATGATTTAA) was purchased from Qiagen (Valencia, CA, USA). Anti-YY2K247me1 and anti-YY2K247pan antibodies were generated by GenScript, Inc (Piscataway, NJ, USA). Antigen (peptide sequence) used for generating anti-YY2K247me1 and anti-YY2 K247pan was CTKVKPKRSK(me1)GEPPK; anti-Flag (F1804) antibody was purchased from Sigma; anti-SET7/9 (07–314) was purchased from Upstate (Billerica, MA, USA); anti-LSD1/AOF2 (A300-215A) was purchased from Bethyl Laboratory Inc. (Montgomery, TX, USA); anti-HA (ab9110) used for ChIP-seq was purchased from Abcam (Cambridge, MA, USA); anti-GAPDH (sc-25778) and anti-ACTIN (SC-8432) were purchased from Santa Cruz Biotechnology (Santa cruz, CA, USA). Peptide sequences were as follows: YY2 K247: CTKVKPKRSKGEPPK; YY2K247me1: CTKVKPKRSK(me1)GEPPK; YY2 K139: TSTQSRSKKPSKKPS.

### siRNA transfection, RNA isolation and RT-qPCR

siRNA transfections were performed using Lipofectamine 2000 (Invitrogen) according to the manufacturer’s protocol. Total RNA was isolated using RNeasy Mini Kit (Qiagen) following the manufacturer’s protocol. First-strand cDNA synthesis from total RNA was carried out using iScript cDNA Synthesis Kit (Bio-Rad, Hercules, CA, USA), followed by qPCR using Mx3005 machine (Stratagene). All RT-qPCRs were repeated at least three times and representative results were shown. Data were presented as mean±s.e.m. Significance test was performed using Student’s *t*-test. Sequence information for all primers used to check gene expression was presented in Supplementary Table S5.

### Plasmid transfection, lentivirus packaging and infection, IB and immunoprecipitation

Plasmid transfections were performed using Lipofectamine 2000 (Invitrogen) according to the manufacturer’s protocol. To pack lentivirus, HEK293T cells were seeded in culture plates coated with poly-D-lysine (0.1% (w/v), Sigma, P7280) and transfected with pBobi-flag-YY2-HA together with packaging vectors, pMDL, VSVG and REV, at a ratio of 10:5:3:2 using Lipofectamine 2000 for 48 h. Virus was collected, filtered and added to HeLa cells in the presence of 10 μgml^−1^ polybrene (Sigma, H9268), followed by centrifugation for 30 min at 1 500 *g* at 37 °C. Medium was replaced 12 h later. IB and immunoprecipitation were performed following the protocol described previously [63, 64].

### Purification of bacterially expressed or HeLa-overexpressed proteins

His-tagged proteins were expressed in BL21 (DE3) bacterial cells (Stratagene) and purified by using Ni-NTA agarose (Qiagen), following the protocol described previously [63, 64]. To purify Flag-tagged proteins from HeLa cells, cells were lysed in lysis buffer containing 50 mM Tris-HCl, pH 7.4, 150 mM NaCl, 1 mM EDTA, 1% Triton X-100. Flag-tagged proteins were then affinity-purified by using anti-Flag M2 agarose and were washed extensively with washing buffer (the same as lysis buffer) before elution with 3XFlag peptides (Sigma).

### *In vitro* methylation and demethylation assay

*In vitro* methylation assay was performed by mixing purified bacterially expressed YY2 full length, truncations or point mutations with histone lysine methyltransferases in methylation buffer (50 mM Tris-HCl, pH 8.0, 20 mM KCl, 5 mM DTT (dithiothreitol), 4 mM EDTA) in the presence of 2 μCi L-[*methyl*-^3^H]-methionine at 37 °C for 1 h. The reaction was stopped by adding sodium dodecyl sulphate (SDS) sample buffer followed by SDS-polyacrylamide gel electrophoresis gel and autoradiogram. For *in vitro* methylation assay using peptides as substrates, enzyme (SET7/9) in the reaction was removed by adding Ni-NTA agarose before dot blot assay. If MALDI-TOF was used to analyze methylation on peptides, the reaction was subjected to desalting and MALDI-TOF as described below. *In vitro* demethylation assay was performed by adding purified bacterially expressed wt LSD1 or its enzymatically dead mutant (m) to pre-methylated YY2 protein or peptide in demethylation buffer (50 mM Tris pH 8.5, 50 mM KCl, 5 mM MgCl_2_, 0.5% bovine serum albumin and 5% glycerol) at 37 °C for 4 h. For *in vitro* demethylation assay using peptides as substrates, enzyme (LSD1 (wt) or LSD1 (m)) was removed by adding Ni-NTA agarose before dot blot assay. If MALDI-TOF was used to analyze demethylation on peptides, the reaction was subjected to desalting and MALDI-TOF as described below.

### MALDI-TOF MS analysis

*In vitro* methylation or demethylation reactions were first desalted with ZipTip (C18; Millipore, Billerica, MA, USA) according to the manufacturer’s protocol with minor modifications. Briefly, ZipTip that was pre-washed with 100% ACN was equilibrated 10 times with 0.1% formic acid (FA). Samples were adjusted to pH 2–3 using FA, and then loaded on ZipTip by slowly aspirating and dispensing for 10 times, followed by washing with 0.1% FA for 10 times. The peptides were finally eluted using 70% ACN with 0.1% FA. MALDI-TOF MS analyses were performed on a Bruker Autoflex II mass spectrometer (Bruker Daltonics, Billerica, MA, USA) in a positive reflection mode. The mass spectrometer was equipped with a pulsed nitrogen laser operated at 337 nm with 3 ns duration pulses and employed stainless steel targets (MTP 384 target ground steel, Bruker Daltonics). Voltage impressed on the ion one and two was 20.0 and 19.0 kV, respectively. The laser power energy was adjusted as needed. The acceleration voltage, grid voltage and delayed extraction time were set as 19 kV, 90% and 150 ns, respectively. 2,5-Dihydroxybenzoic acid (Sigma) matrix was prepared at a concentration of 20 mgml^−1^ in ACN/water (1:1, v/v) with 0.1% FA. Samples were prepared by applying 1 μl mixture solution (1:1, v/v) of sample and matrix onto the stainless steel MALDI target plate, allowing the droplet to dry in the air at room temperature (RT) before transferring into mass spectrometer.

### In-gel digestion and LC-MS/MS analysis

The gel slices were cut to cubes (1×1 mm) and transferred to Lobind tubes (Eppendorf), and 300 μl liquid chromatography–mass spectrometry (LC-MS) water was then added for 15 min at RT with agitation. Same volume of ACN was added and incubated for 15 min. The supernatant was discarded and 100 μl LC-MS ACN was then added for 5 min at RT. Samples were dried in a Speedvac (Eppendorf) and then reduced by mixing with 200 μl of 100 mM ammonium bicarbonate/10 mM DTT and were incubated at 56 °C for 30 min. The liquid was removed and 200 μl of 100 mM ammonium bicarbonate/50 mM iodoacetamide was added to gel pieces and incubated at RT in the dark for 30 min. After removal of the supernatant and one wash with 300 μl 100 mM ammonium bicarbonate for 15 min, same volume of ACN was added to dehydrate the gel pieces. The solution was then removed and samples were dried in a Speedvac. For digestion, enough solution of ice-cold trypsin (0.01 μgμl^−1^) in 20 mM ammonium bicarbonate was added to cover the gel pieces and then set on ice for 30 min. After complete rehydration, the excess trypsin solution was removed, replaced with 20 mM ammonium bicarbonate to completely cover the gel pieces and left overnight at 37 °C. The peptides were extracted twice with 50 μl of 50% ACN/1% FA and by vortex-mixing at RT for 30 min. All extracts were pooled and dried in a Speedvac, followed by using ZipTips to purify and concentrate peptides for LC-MS/MS analysis.

MS experiments were performed on a nanoscale UHPLC system (EASY-nLC1000, Proxeon Biosystems, Odense, Denmark) connected to an Orbitrap Q-Exactive equipped with a nanoelectrospray source (Thermo Fisher Scientific, Waltham, MA, USA). The peptides were dissolved in 0.1% FA with 2% ACN and separated on a reverse-phase high-performance liquid chromatography (RP-HPLC) analytical column (75 μm×15 cm) packed with 2 μm C18 beads (Thermo Fisher Scientific) using a 2 h gradient ranging from 5 to 35% ACN in 0.1% FA at a flow rate of 300 nl/min. The spray voltage was set at 2.5 kV and the temperature of ion transfer capillary was 275 °C. A full MS/MS cycle consisted of one full MS scan (resolution, 70 000; automatic gain control value (AGC), 1e6; maximum injection time, 100 ms) in a profile mode over a mass range between *m*/*z* 350 and 1 800, followed by fragmentation of the top 20 most intense ions by high-energy collisional dissociation with normalized collision energy at 28% in centroid mode (resolution, 17 500; AGC value, 1e; maximum injection time, 100 ms). The dynamic exclusion window was set at 30 s. One microscan was acquired for each MS and MS/MS scan. Unassigned ions or those with a charge of 1+ and >7+ were rejected for MS/MS. Raw data were processed using Proteome Discoverer (version 2.1), and MS/MS spectra were searched against the reviewed Swiss-Prot human proteome database. All searches were carried out with precursor mass tolerance of 10 p.p.m., fragment mass tolerance of 0.02 Da, oxidation (Met; +15.9949 Da), methylation (Arg, Lys; +14.0266 Da), dimethylation (Arg, Lys; +28.0532 Da) and acetylation (protein N terminus) (+42.0106 Da) as variable modifications, carbamidomethylation (+57.0215 Da) as fixed modification and two trypsin missed cleavages allowed. Only peptides with at least six amino acids in length were considered. The peptide and protein identifications were filtered by Proteome Discoverer to control the false discovery rate <1%. At least one unique peptide was required for protein identification.

### Electrophoretic mobility shift assay

EMSA was performed by using the LightShift Chemiluminescent EMSA Kit (20148) from Pierce (Rockford, IL, USA) following the same protocol as described previously [37].

### Establishing inducible HeLa cells stably expressing pRevTRE-Flag-YY2-HA

HeLa cells stably expressing pTet-On-Advanced (Clontech, Palo Alto, CA, USA) were transfected with pRevTRE-Flag-YY2-HA, and then selected with hygromycin (200 μgml^−1^). To induce the expression of YY2, doxycycline was added at a final concentration of 600 ng/ml for 48 h.

### Generation of YY2, SET7/9 or LSD1/AOF2 KO cell lines using CRISPR/Cas9 gene-editing technology

SET7/9 KO HeLa cells generated by using CRISPR/Cas9 system were described previously [37]. YY2 or LSD1/AOF2 KO HeLa cells were generated using similar approaches. Specifically, gRNA sequence targeting YY2 (5′- TCGTACTGGACGCTTTCCGTCGG-3′) or LSD1 (5′- TCGGACCAGCCGGCGCAAGCGGG-3′) was cloned into gRNA cloning vector (Addgene, 41824, Cambridge, MA, USA) and confirmed by sequencing. To screen for YY2 or LSD1 KO clones, HeLa cells were transfected with pcDNA3.3-hCas9 (Addgene, 41815, Cambridge, MA, USA) and gRNA expression vectors, followed by G418 selection (0.5 mgml^−1^). Single colonies were subjected to IB to select KO ones, which were further validated by PCR using genomic DNA as template followed by Sanger sequencing. The sequencing information for primer sets used was as follows: YY2: Forward (F) 5′- TGGCCTCCAACGAAGATTTC-3′ and Reverse (R) 5′- GCATTTCCTGGTCGTGGTC-3′; LSD1/AOF2: Forward (F) 5′- GCAAGAAAGAGCCTCCGC-3′ and Reverse (R) 5′- GTGTCGTTTGAGGGAAGGGT-3′.

### Chromatin immunoprecipitation

ChIP was performed following the protocol described previously [63, 64]. Briefly, cells were fixed with 1% formaldehyde (Sigma) for 10 min at RT. Fixation was stopped by adding glycine (0.125 M) and incubated for 5 min at RT, followed by washing with phosphate-buffered saline (PBS) twice. Chromatin DNA was sheared to 300~500 bp average in size through sonication. Resultant was immunoprecipitated with control IgG or anti-HA antibody (ab9110) overnight at 4 °C, followed by incubation with protein G magnetic beads (Invitrogen) for additional 2 h. After washing and elution, the protein–DNA complex was reversed by heating at 65 °C overnight. Immunoprecipitated DNA was purified by using QIAquick spin columns (Qiagen) and analyzed by qPCR using Mx3005 machine (Stratagene). All ChIP–qPCRs were repeated at least three times and representative results were shown. Sequence information for all primers used for ChIP was presented in Supplementary Table S5.

### ChIP coupled with high-throughput sequencing

ChIP-seq sample preparation and computational analysis of ChIP-seq data were performed as described previously [37]. YY2 ChIP-seq data were deposited in the Gene Expression Omnibus database under accession GSE76856.

The following link has been created to allow review of record GSE76856 while it remains in private status: http://www.ncbi.nlm.nih.gov/geo/query/acc.cgi?token=edqxuisoxdsxrwt&acc=GSE76856.

### RNA sequencing

Total RNA was isolated from HeLa cells using RNeasy Mini Kit (Qiagen) following the manufacturer’s protocol. DNase I in column digestion was included to ensure the RNA quality. RNA library preparation was performed by using NEBNext Ultra Directional RNA Library Prep Kit for Illumina (E7420L) (San Diego, CA, USA). Paired-end sequencing was performed with Illumina HiSeq 3000 at RiboBio Co., Ltd (Guangzhou, China). For computational analysis of RNA-seq data, sequencing reads were aligned to hg19 human genome using the 'Tuxedo suite': Bowtie2-Tophat [65]. EdgeR was used to compute the differential expressed genes (*P*<0.001, dispersion 0.001) [66]. The HOMER suite (Hypergeometric Optimization of Motif EnRichment) was used to generate the input of EdgeR and call EdgeR [67]. GO analysis for genes regulated by YY2 was done using David [68]. RNA-seq data were deposited in the Gene Expression Omnibus database under accession GSE96877.

The following link has been created to allow review of record GSE96877 while it remains in private status: https://www.ncbi.nlm.nih.gov/geo/query/acc.cgi?token=oxqpgqkexfqpbaz&acc=GSE96877.

### Cell proliferation assay, colony formation assay and tumor xenograft assay

A total of 2×10^5^ of HeLa cells were seeded in one well in a six-well plate after infection with lentiviral particles for 48 h, and cell number was counted every 2 days. After each counting, 2×10^5^ cells were seeded for the next count. For colony formation assay, 2 000 cells infected with lentiviral particles were seeded in one well in a six-well plate, and colony formation was examined 10 days after. Briefly, colonies were fixed with methanol/acid solution (3:1) for 5 min and stained with 0.1% crystal violet for 15 min. For quantification, the crystal violet dye was released into 10% acetic acid and measured at wavelength 590 nm (OD590). For tumor xenograft assay, 1.5 million HeLa cells infected with lentiviral particles were suspended in PBS for subcutaneous injection into female athymic Nu/Nu mice between the ages of 28 and 42 days. All mice were killed 3 weeks after subcutaneous injection. Tumors were then excised, photographed and weighed. Animals were housed in the Animal Facility at Xiamen University under pathogen-free conditions, following the protocol approved by the Xiamen Animal Care and Use Committee.

### Immunofluorescence

HeLa cells were fixed with 4% paraformaldehyde in PBS for 20 min, and then permeabilized with 0.1% Triton X-100 in PBS on ice for 10 min. After rinsing with PBS buffer for three times, blocking solution (1% bovine serum albumin in PBS) was applied for 1 h and primary antibodies against Flag (Sigma,1:200) and SET7/9 (Upstate,1:200) were added in blocking buffer at 4 °C overnight. After washing with PBS/0.1% Triton X-100 for five times, cells were incubated with DAPI (4',6-Diamidino-2-phenylindole) and secondary antibodies conjugated with fluorescent dyes for 1 h, washed with PBS/0.1% Triton X-100, and mounted in Fluoromount-G (Southern Biotech, Birmingham, AL, USA). Images were recorded on a ZEISS Exciter 5 microscope (ZEISS, Jena, Germany).
