# Supplementary material for: Methylation of transcription factor YY2 regulates its transcriptional activity and cell proliferation
Source: Cell Discov. 2017 Oct 3;3:17035–. doi: 10.1038/celldisc.2017.35 (PMC5665210; doi:10.1038/celldisc.2017.35)
Supplement: Supplementary Figures [file celldisc201735-s1.pdf]

Supplementary Information

Supplementary Figure Legends

Figure S1. SET7/9 methylates YY2.

(A, B) *In vitro* methylation assay was performed by using SET7/9 alone from bacterial cells (A) or HEK293T cells with over-expression (B), followed by autoradiogram. Stars indicate automethylation (auto-me) of SET7/9.

(C) Human YY2 sequence. YY2 amino-terminus (YY2 (aa1-226)) (blue); YY2 carboxyl-terminus (YY2 (aa227-372)) (black); Zn fingers (in parentheses). Lysine residues (K139, K247 and K369) embedded in SET7/9 consensus methylation sites were underlined (red).

(D) *In vitro* methylation assay was performed by mixing synthetic short peptides from YY2 containing unmodified or mono-methylated K247 (YY2K247me1) with or without purified bacterially-expressed SET7/9 protein, followed by MALDI-TOF MS analysis.

(E) *In vitro* methylation assay was performed by mixing synthetic short peptides from YY2 containing unmodified K139 with or without purified bacterially-expressed SET7/9 protein, followed by MALDI-TOF MS analysis.

(F) Sequence alignment of the region surrounding lysine 247 (K247) (boxed) in paralogous YY2 genes in various organisms by using Clustal Omega. Asterisk (*) indicates positions which have fully conserved residue; Colon (:) indicates conservation between groups of strongly similar properties; Period (.) indicates conservation between groups of weakly similar properties. *Hs, Homo sapiens*; *Pt, Pan troglodytes*; *Pp, Pan paniscus*; *Nl, Nomascus leucogenys*; *Mf, Macaca fascicularis*; *Gg, Gorilla gorilla*; *Sb, Saimiri boliviensis*; *Pa, Papio anubis*; *Cj, Callithrix jacchus*; *Cs, Chlorocebus sabaeus.*

(G) Sequence alignment of human YY1 and YY2 by using Clustal Omega. Boxed residue is lysine 247 (K247) in YY2 and its homologous lysine 288 (K288) in YY1.

Figure S2. Effects of SET7/9 or LSD1 on YY2 K247 methylation.

(A) HeLa cells transfected with control siRNA or siRNA specifically targeting *SET7/9* or *LSD1* in the presence of YY2 were subjected to IB with antibodies as indicated.

(B) Genomic DNA was extracted from LSD1 knock-out (KO) cells generated by CRISPR/Cas9 system, followed by PCR using specific primer sets surrounding gRNA targeting region (boxed in blue). The resultant PCR products were subjected to Sanger sequencing and genomic region with deletions was shown, with one or ten basepair (bp) removed as shown in dash. Translation start codon (ATG) (dark red).

Figure S3. SET7/9-mediated YY2 K247 methylation does not alter YY2 cellular localization.

(A) Control (wt) or SET7/9 knockout (KO) HeLa cells transfected with vector expressing Flag-tagged YY2 were subjected to immunofluorescence (IF) analysis as indicated.

(B) HeLa cells transfected with vector expressing Flag-tagged YY2 and SET7/9 were subjected to IF analysis as indicated.

(C) HeLa cells transfected with vector expressing Flag-tagged YY2 (wt) or YY2 (K247R) were subjected to IF analysis as indicated.

Figure S4. YY2 K247 methylation regulates YY2 binding with chromatin.

(A) HeLa cells were subjected to ChIP analysis as described in Figure. 5D. ChIP signals were presented as fold induction over wt after normalized to input (± s.e.m., $^{*}P<0.05$, $^{**}P<0.01$).

(B) The expression of YY2 (wt) and YY2 (K247R) as described in Figure. 5D and (A) was examined through IB.

(C) Control (wt), SET7/9 or LSD1 knockout (KO) HeLa cells were subjected to ChIP analysis as described in Figure. 5E. ChIP signals were presented as fold induction over wt after normalized to input (± s.e.m., **P<0.01, ***P<0.001).

(D) The expression of YY2 as described in Figure. 5E and (C) was examined through IB.

(E) The expression of YY2, SET7/9 (wt), SET7/9 (m), LSD1 (wt) and LSD1 (m) as described in Figure. 5F was examined by IB using antibodies as indicated.

Figure S5. YY2 K247 methylation is involved in YY2-regulated gene transcription.

(A) Genomic DNA was extracted from YY2 knock-out (KO) cells generated by CRISPR/Cas9 system, followed by PCR using specific primer sets surrounding gRNA targeting region (boxed in blue). The resultant PCR products were subjected to Sanger sequencing and genomic region with deletions was shown, with seven basepair (bp) removed as shown in red. Translation start codon (ATG) (dark red).

(B) HeLa cells were transfected with luciferase reporter containing YY1 consensus binding site (pGL2-YY1-*luc*) in the presence or absence of control vector or vectors expressing YY2(wt) or YY2 (K247R), followed by luciferase reporter activity measurement (± s.e.m., ***P<0.001).

(C) The expression of YY2 (wt) and YY2 (K247R) as described in (B) was examined through IB with anti-Flag antibody.

Figure S6. YY2 somatic mutations alter YY2 binding with chromatin.

(A, D, E) The expression of YY2 (wt), YY2 (K244Q) or YY2 (S246F) as described in Figure. 7C (A), Figure. 7D (D) and Figure. 7E (E) was examined through IB with anti-Flag antibody.

(B) DNA EMSA assay was performed by incubating biotinylated oligonucleotide containing YY1 consensus binding site with *in vitro* purified bacterially-expressed YY2 (wt), YY2 (K244Q)

or YY2 (S246F). The expression of YY2 (wt), YY2 (K244Q) or YY2 (S246F) was examined through C.B.S (bottom panel).

(C) HeLa cells were subjected to ChIP as described in Figure. 7D. ChIP signals were presented as fold induction over wt after normalized to input (± s.e.m., **$P<0.01$, ***$P<0.001$).

Figure S7. YY2 K247 methylation is involved in YY2-regulated cell proliferation and tumor growth.

(A) HeLa cells as described in Figure. 8A were seeded for colony formation, and cell colonies were fixed and stained with crystal violet.

(B) Quantification of the crystal violet dye as shown in (A). (n=3, ± s.e.m., ***$P<0.001$)

Supplementary Table Legend

Table S1. MS2 spectrum of K139-methylated peptides in YY2. *In vitro* methylation assay was performed by mixing synthetic short peptide containing YY2 K139 with SET7/9 followed by LC MS/MS analysis. MS2 spectrum of K139-methylated peptide was shown**.**

Table S2. MS2 spectrum of K247-methylated peptides in YY2 *in vitro*. *In vitro* methylation assay was performed by mixing *in vitro* purified bacterially-expressed YY2 and SET7/9 followed by mass spectrometry (MS) analysis. MS2 spectrums of peptides containing K247-methylation as well as the corresponding unmodified peptides were shown.

Table S3. MS2 spectrum of K247-methylated peptides in YY2 in cultured cells. HeLa cells were transfected with Flag-tagged YY2 and SET7/9, followed by affinity purification and MS analysis. MS2 spectrum of peptide containing K247-methylation as well as the corresponding unmodified peptide was shown.

Table S4. Gene ontology analysis for genes regulated by YY2, SET7/9 or LSD1. Gene ontology analysis for genes regulated, both positively and negatively, by YY2, SET7/9 or LSD1,

genes positively regulated by YY2 and SET7/9 in common, and genes positively-regulated by YY2 and SET7/9 in common and negatively-regulated by LSD1 was done using David[68].

Table S5. Sequence information for all qPCR primers used in the current study. Sequence information of qPCR primers specifically targeting to pGL2-*luc* vector, *TP53/p53*, *RAD1*, *ABL1*, *CCNT2* or *CCNA2* gene promoter regions to examine YY2 binding after ChIP, and primers specifically targeting to *OLR1*, *PDE2A*, *MYPN*, or *PTGS2* to examine their expression levels after reverse transcription (RT) were shown. F: forward; R: reverse.

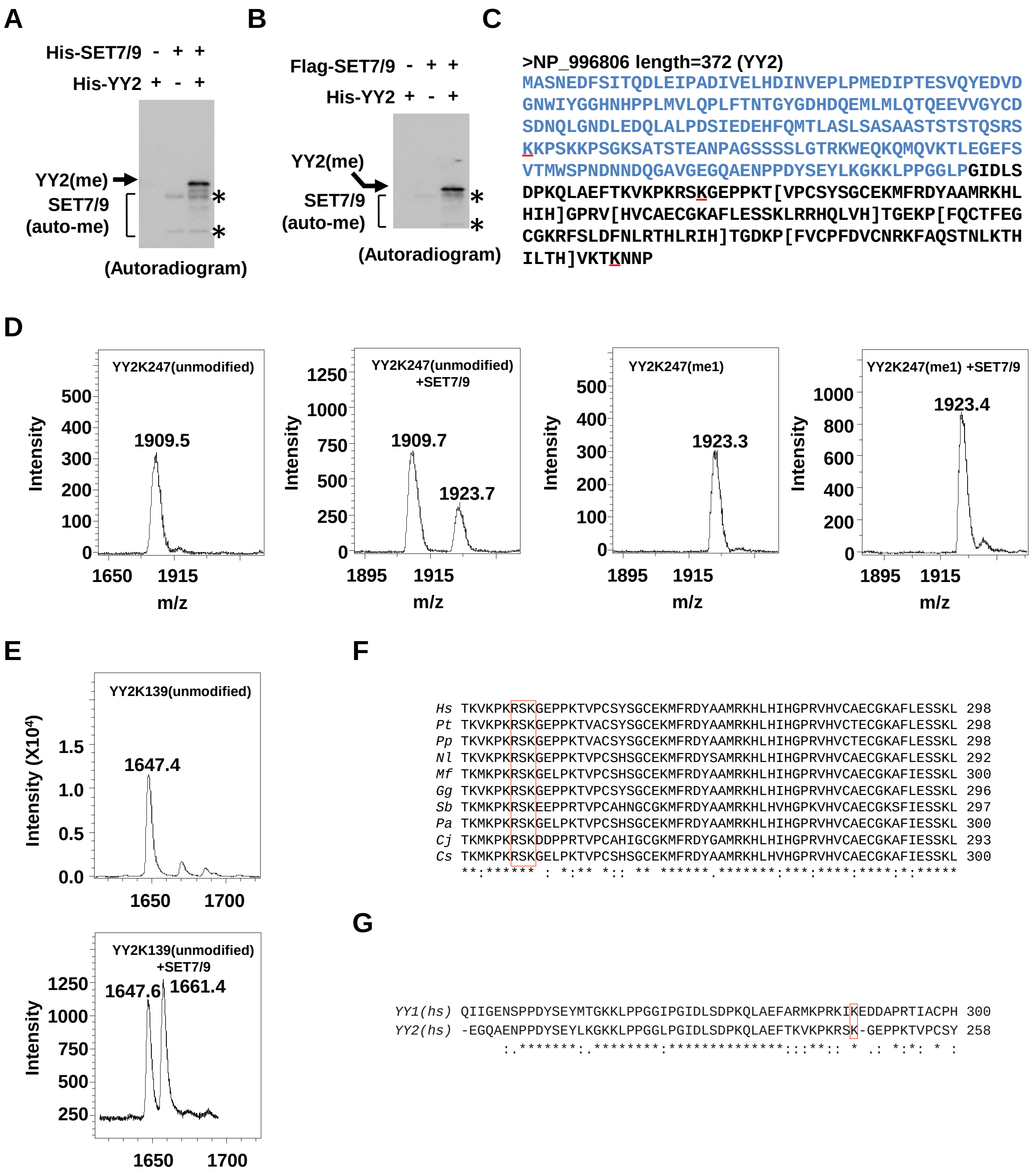

Figure S1 (Liu)

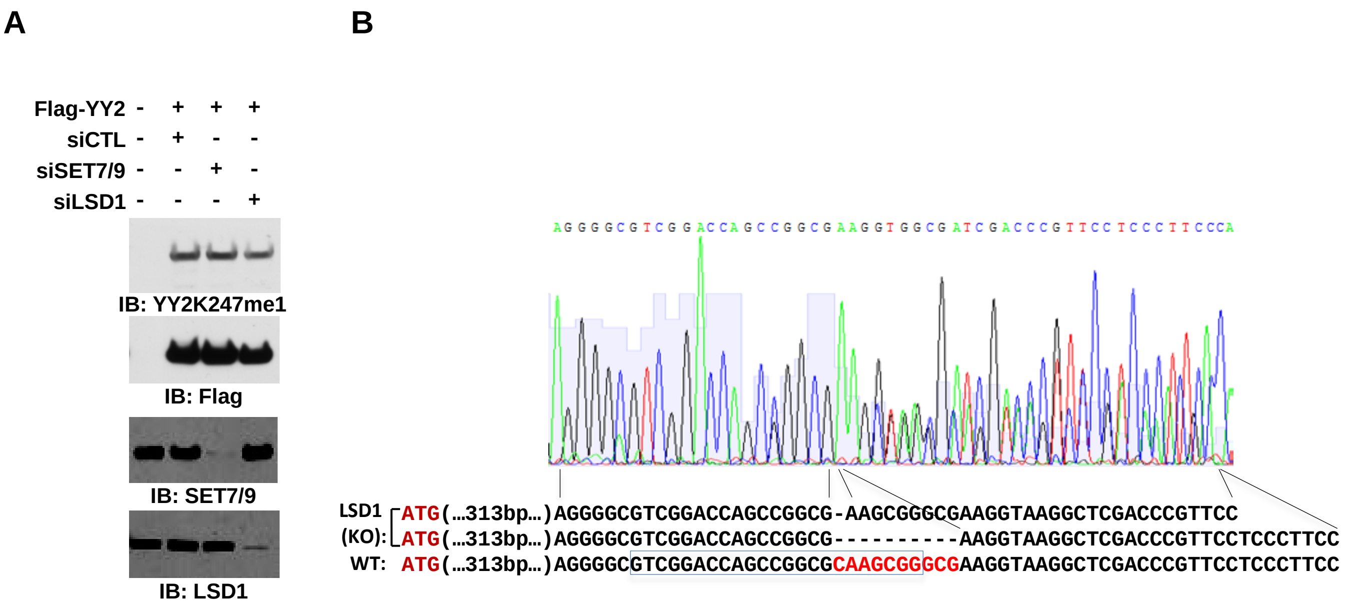

Figure S2 (Liu)

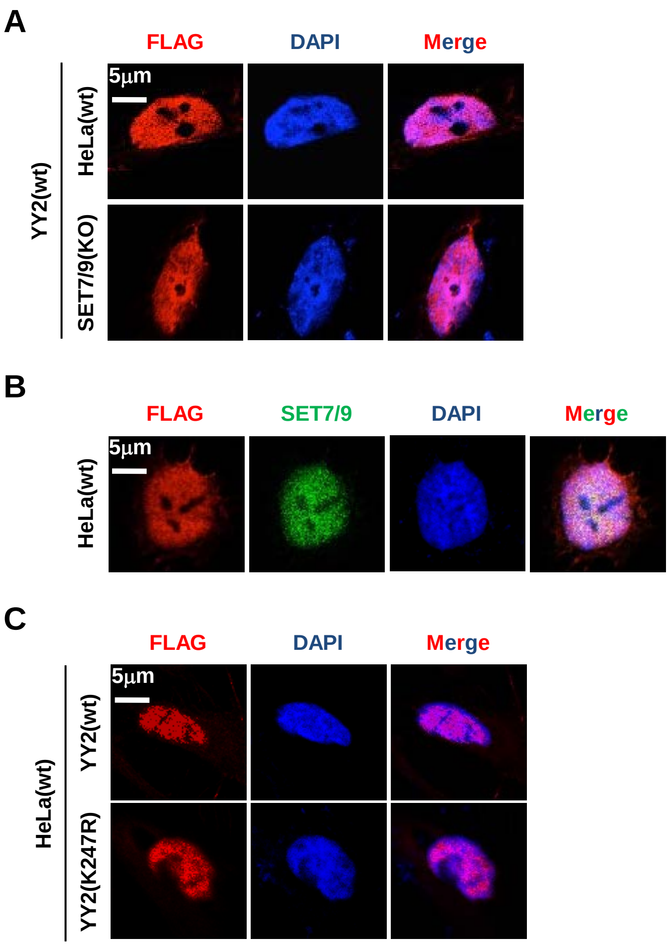

Figure S3 (Liu)

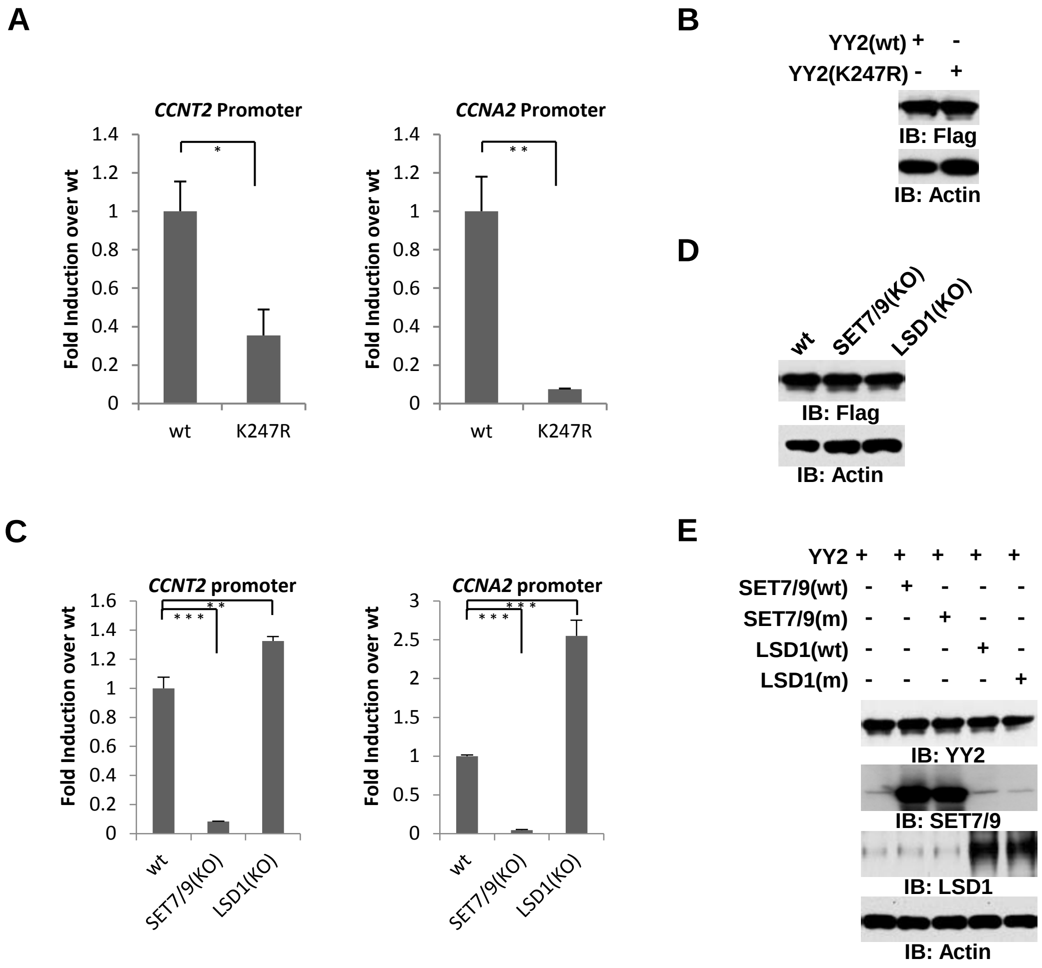

Figure S4 (Liu)

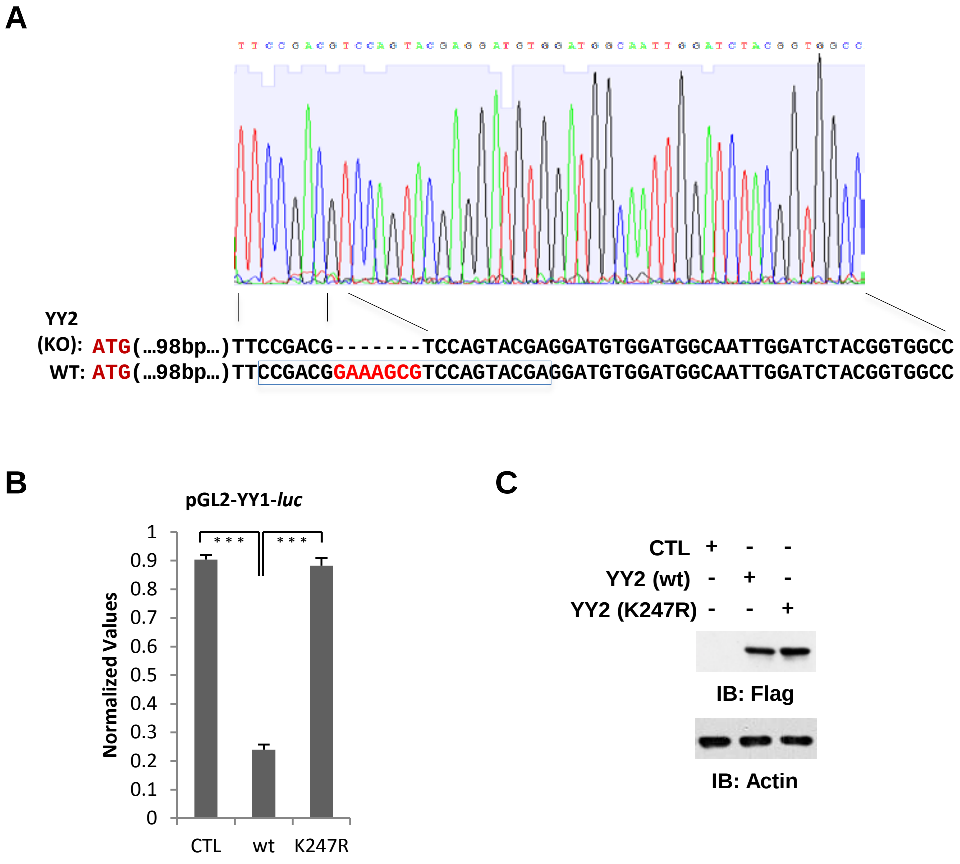

Figure S5 (Liu)

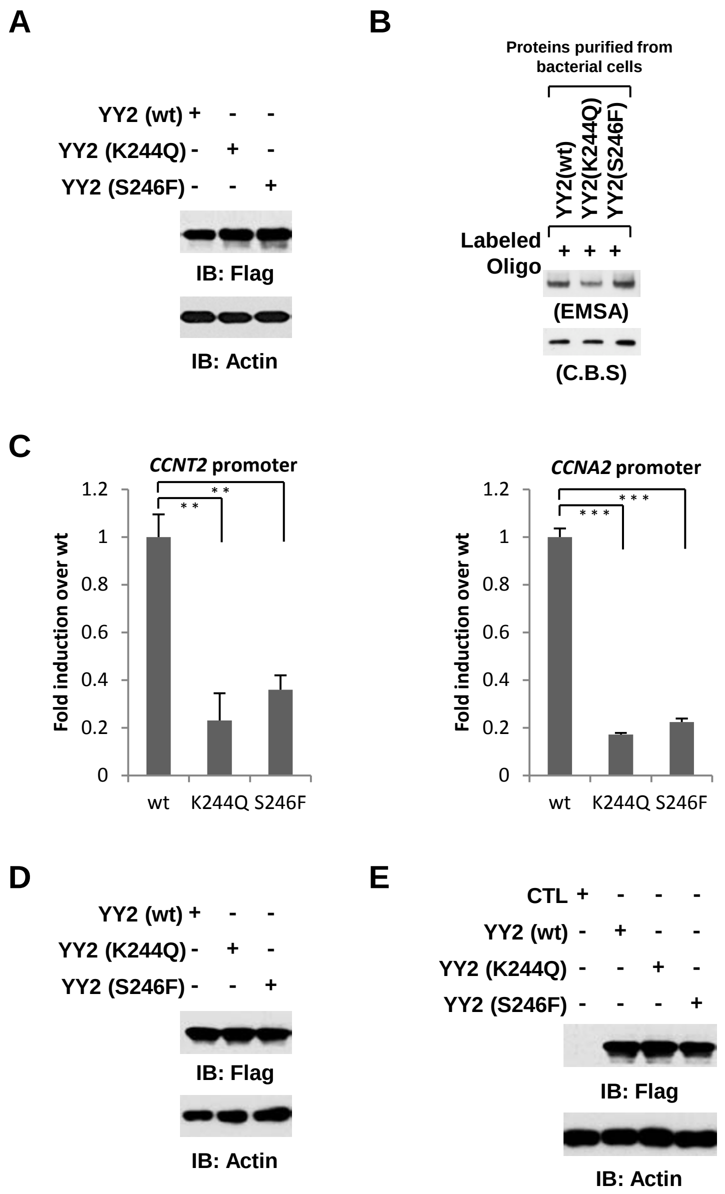

Figure S6 (Liu)

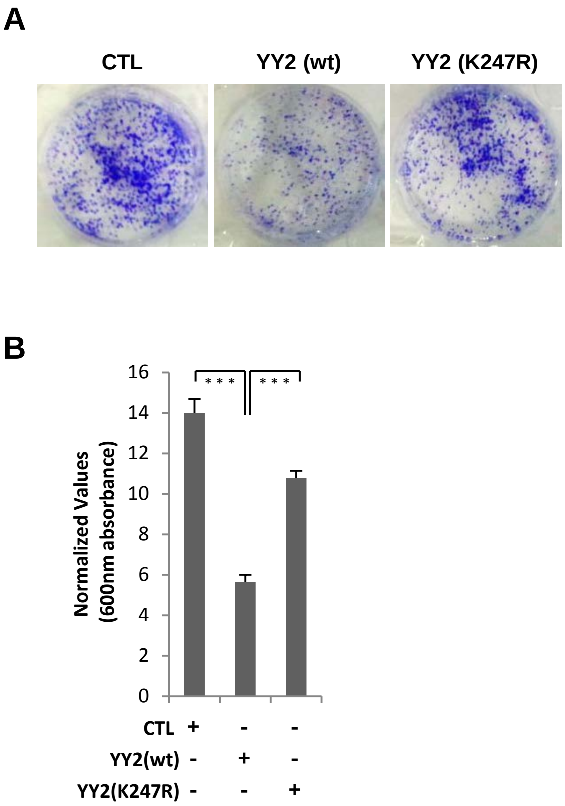

Figure S7 (Liu)
